# From a bistable adsorbate to a switchable interface: tetrachloropyrazine on Pt(111)†

**DOI:** 10.1039/d1nr07763e

**Published:** 2022-03-10

**Authors:** Lukas Hörmann, Andreas Jeindl, Oliver T. Hofmann

**Affiliations:** Institute of Solid State Physics, Graz University of Technology Petersgasse 16 8010 Graz Austria o.hofmann@tugraz.at +43 316 873 8466 +43 316 873 8964

## Abstract

Virtually all organic (opto)electronic devices rely on organic/inorganic interfaces with specific properties. These properties are, in turn, inextricably linked to the interface structure. Therefore, a change in structure can introduce a shift in function. If this change is reversible, it would allow constructing a switchable interface. We accomplish this with tetrachloropyrazine on Pt(111), which exhibits a double-well potential with a chemisorbed and a physisorbed minimum. These minima have significantly different adsorption geometries allowing the formation of switchable interface structures. Importantly, these structures facilitate different work function changes and coherent fractions (as would be obtained from X-ray standing wave measurements), which are ideal properties to read out the interface state. We perform surface structure search using a modified version of the SAMPLE approach and account for thermodynamic conditions using *ab initio* thermodynamics. This allows investigating millions of commensurate as well as higher-order commensurate interface structures. We identify three different classes of structures exhibiting different work function changes and coherent fractions. Using temperature and pressure as handles, we demonstrate the possibility of reversible switching between those different classes, creating a dynamic interface for potential applications in organic electronics.

## Abstract 2

Tetrachloropyrazine on Pt(111) forms three different classes of interfaces structures with different work functions and coherent fractions. We show that these structures can be switched using temperature and/or pressure.

## Graphical abstract

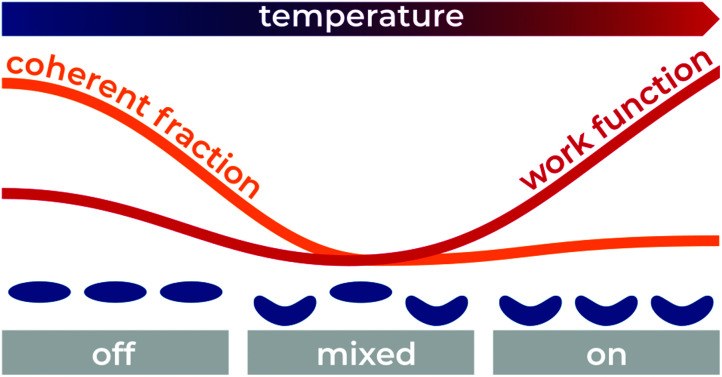

## 1 Introduction

Organic/inorganic interfaces are essential for the construction of organic (opto)electronic devices. To date research has mainly focused on attaining specific interface properties through controlling the structure and chemistry of the organic adlayer.^1–5^ In this theoretical study we go beyond these efforts and study organic adsobate layers with switchable properties.

Switchable interfaces in the literature rely on several function principles including modifying interactions at the interface^6^ and applying external stimuli such as optical signals,^7,8^ electric fields,^9,10^ magnetic fields,^11^ temperature,^12^ biochemical processes,^13^ or pH-value.^14^ Here, we use temperature and pressure to switch adlayers of molecules that exhibit a double-well potential when adsorbing on a substrate. Examples for systems with double-well potentials include benzene derivatives on Pt(111),^15–17^ tetrafluoropyrazine on Ni(111)^18^ and anthradithiophene on Cu(111).^19–21^ Related examples are molecules adopting different conformers upon adsorption.^22^ These systems show great potential as molecular switches^16^ and have even been suggested as data storage.^23^ Here, we focus on the system of tetrachloropyrazine (TCP) on Pt(111), which displays a double-well potential on the Pt(111) surface.^16^ The two types of adsorption geometries, called on- and off-state, exhibit different types of bonding and adsorption geometries. Crucially, both minima have approximately the same adsorption energy, allowing the formation of diverse surface structures which are sufficiently close in energy to be reversibly switched with external stimuli. Interestingly, in this work we find that individual chemisorbed molecules are energetically more favourable than physisorbed ones. However, this ordering reverses in continuous layers (at low temperatures).

Reading out the interface states requires that the switch in structure entails a shift in interface properties. We focus on two of these properties, namely the work-function change Δ*Φ* (relative to the clean surface) and the coherent fraction (as obtained by X-ray standing wave measurements), and study how to modify them using environmental stimuli such as temperature and pressure. Different possible polymorphs exhibit diverse Δ*Φ*s and coherent fractions. However, only a fraction of the potential range for work functions can be thermodynamically accessed. We identify the reasons for this and provide ideas to overcome it.

Molecules on surfaces may arrange into a large number of possible polymorphs. Therefore, we investigate the Δ*Φ*s and the coherent fractions of millions of surface structures. To determine these structures and their adsorption energies we use the SAMPLE approach,^24^ which combines dispersion-corrected density functional theory (DFT) with machine learning (see Methods section). Further, *ab initio* thermodynamics allows modelling the impact of temperature and pressure on the surface polymorphism. This enables us to conduct *in silico* simulations of experiments.

A number of experimental methods exist to determine work functions (and in turn Δ*Φ*s), such as photo-emission spectroscopy and the Kelvin probe method. The change in work function resulting from molecules adsorbing on a surface depends on a number of factors. These include pushback of electron density into the surface, charge transfer across the interface, formation of new interface states and bonds between adsorbates and the substrate, image charge effects, permanent dipoles of the molecules as well as their coverage on the surface.^25–27^ On- and off-state differ significantly in the way they bond to the surface and their adsorption geometry. Therefore, we expect that adsorbate layers consisting of on- and off-state adsorption geometries yield dissimilar Δ*Φ*s, allowing the construction of a switchable interface.

A useful experimental method to determine the adsorption geometry is the X-ray standing wave (XSW) technique.^28^ This method yields two measures, namely the coherent position and the coherent fraction. The coherent position allows determining the mean adsorption height of the adsorbates. The coherent fraction is effectively an order parameter^29^ containing information about differences in adsorption heights, which would occur if on- and off-state molecules coexisted in a particular interface state. If all atoms (of a particular species) within all molecules adsorb at an identical adsorption height, the coherent fraction is 1, while it decreases with variations in adsorption height. Thus, the coherent fraction will allow differentiating between on-, off- and mixed-state layers.

The fact that both Δ*Φ* and the coherent fraction are experimentally readily accessible makes these properties ideal candidates to readout the state of a switchable interface.

## 2 Results

### 2.1 Individual molecules on the surface

When investigating the adsorption of single molecules, we are interested in the local minima of the potential energy surface (PES), which constitute energetically favourable adsorption geometries. We will hereafter refer to these as “geometries”. As stated above, TCP on Pt(111) exhibits a double-well potential, with minima occurring at two different adsorption heights. Hence, two different types of adsorption geometries exist, namely on- and off-state geometries. While on-state geometries are chemisorbed, off-state geometries are physisorbed.

We determine the geometries in two steps: initially, a Gaussian-process regression (GPR) algorithm identifies the minima geometries of a coarse-grained PES (see Methods section). The algorithm uses DFT-calculated energies of a few adsorption geometries as input and interpolates between them. Hereby, it only considers the most important degrees of freedom, *i.e.*, position, orientation and bending of the molecule (see Methods section). If necessary (see below), we refine the GPR minima using DFT geometry optimisations.

**Fig. 1:**
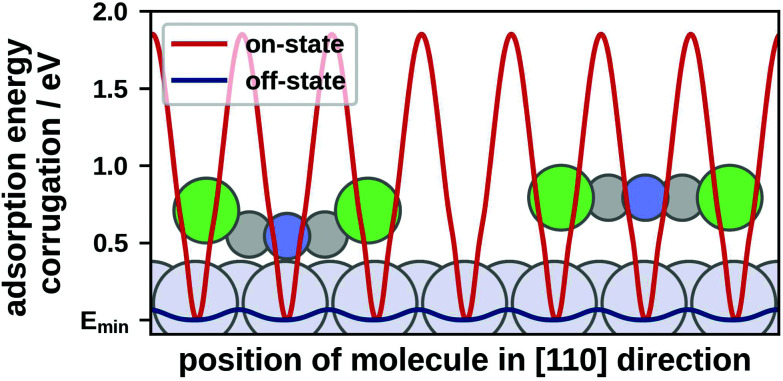
Cut through the GPR PES of TCP on Pt(111) in [110] direction; (red) PES of the on-state geometry; (blue) PES of the off-state geometry; energies are plotted relative to the respective minimum energy *E*_min_.

For off-state geometries the coarse grained PES is sufficiently accurate since the molecules mainly bond *via* spatially uniform van der Waals interactions. This leads to a weakly corrugated PES, with an energy range of approximately 0.2 eV (see Fig. 1). Hence, we can directly use the GPR minima as adsorption geometries. In the off-state the molecules remain flat and have five adsorption geometries with (GPR-calculated) bonding energies of −0.97 eV to −0.95 eV and adsorption heights of approximately 3.3 Å. DFT geometry optimisations of these minima yield only very small changes in geometry (the molecule remains flat) and the gain in adsorption energy is within the uncertainty of our method. The adsorption energies are about 0.10 eV more bonding while the adsorption heights are approximately 0.15 Å higher than previously reported values (−0.88 eV and 3.14 Å).^16,30^ We attribute these deviations to our employment of global structure search (albeit with limited degrees of freedom) and different convergence settings.

Conversely, the PES of on-state geometries is strongly corrugated, with minima and maxima spanning an energy range of approximately 2.0 eV (see Fig. 1). This is due to a pronounced spatial dependence of the covalent interactions between the molecule and the surface. The molecules are strongly bent, with the Cl atoms pointing away from the surface. Due to the higher complexity of the on-state, a PES containing only position, orientation and bending of the molecule is insufficient to accurately determine adsorption geometries. Hence, we refine the GPR minima with DFT, allowing the molecule and the substrate atoms of the two topmost layers to relax. Hereby, the relaxation of substrate atoms below the molecule contributes up to −0.5 eV to the adsorption energies (more negative is more bonding). For the on-state we find four different adsorption geometries that have adsorption energies (calculated with DFT) of −1.07 eV to −1.04 eV and adsorption heights of approximately 2.1 Å above the unrelaxed substrate. (This is how an XSW-experiment would determine the adsorption height.^28^) Similar to the off-state, adsorption energies of the on-state are approximately 0.10 eV more bonding than previously reported values (0.98 eV) while the adsorption heights are in good agreement.^16,30^

For visualisations of the potential energy surfaces and additional details regarding adsorption geometries see the ESI.†

### 2.2 Interface structures

Having discussed the adsorption geometries of individual molecules on the surface, we will now focus on the close-packed layers they form. In general, adlayers assume different types of commensurability depending on a delicate balance between molecule–molecule interactions and the corrugation of molecule–substrate interactions.^31,32^ Strong molecule–molecule interactions and a comparatively weak corrugation of molecule–substrate interactions allow maximising the energy gain from interactions between molecules. This will most likely lead to incommensurate layers. Conversely, a large corrugation of molecule–substrate interactions and comparatively weak molecule–molecule interactions force the molecules to remain in energetically favourable adsorption sites. Concurrently, any energy gain from favourable molecule–molecule interactions would be outweighed by the energy penalty from unfavourable molecule–substrate interactions. This case leads to commensurate layers. Using the arguments from the previous paragraph, we can anticipate if an adlayer will be commensurate or incommensurate.

The two types of local adsorption geometries can form three different classes of adlayer structures. We will hereafter refer to a close-packed adlayer of molecules as a “motif”.

First, off-state motifs consist purely of off-state geometries. Here, the PES of the single molecule is weakly corrugated (see Fig. 1). Hence, the molecule can seek out the most beneficial molecule–substrate interactions, making off-state motifs incommensurate.

Next, on-state motifs consist only of on-state geometries. The PES of on-state geometries is strongly corrugated (see Fig. 1). Therefore, the molecules seek the most favourable molecule–substrate interaction, which leads on-state motifs to be commensurate.

Finally, mixed-state motifs contain both on- and off-state geometries. Here, the off-state geometries seek out the most beneficial molecule–molecule interaction, while the on-state geometries remain stuck due to the strongly corrugated molecule–substrate interactions. Hence, we expect mixed-state motifs to be commensurate.

To find the energetically most favourable motifs, we first perform commensurate structure search using the SAMPLE approach (see Methods section).^24^ Hereby, we generate all possible commensurate motifs with different coverages and up to three molecules per unit cell and predict their adsorption energies. However, as we state above, we expect that some energetically favourable adlayers are incommensurate. Truly incommensurate motifs contain an infinite number of molecules per unit cell, each with a different adsorption site. This makes it obviously impossible to determine their energies from first principles. Nevertheless, we can approximate incommensurate motifs using higher-order commensurability. To consider higher-order commensurate motifs, we use a generalised SAMPLE approach (SAMPLE-GPR) based on a GPR model (see Methods section). This approach also provides energies for geometry optimisations with limited degrees of freedom.

These additional capabilities are necessary to describe higher-order commensurate off-state motifs and to optimise molecule positions in mixed-state motifs, while the SAMPLE approach would be sufficient for on-state motifs. For a consistent treatment of all three motif classes, we rerank the 1000 energetically most favourable motifs of every type and considered coverage (in total approximately 37 000 motifs) *via* SAMPLE-GPR and consider only SAMPLE-GPR energies hereafter.

For the off-state we expect that incommensurate motifs are most energetically favourable. Hence, we use SAMPLE-GPR to optimise the 20 most energetically favourable motifs using the unit cell parameters as well as the location and orientation of the molecules as degrees of freedom (see Methods section).

In mixed-state motifs the physisorbed molecules can move relatively freely. Therefore, we optimise their positions and orientations in the 40 energetically most favourable mixed-state motifs of every coverage, while keeping the chemisorbed molecules fixed.

**Fig. 2:**
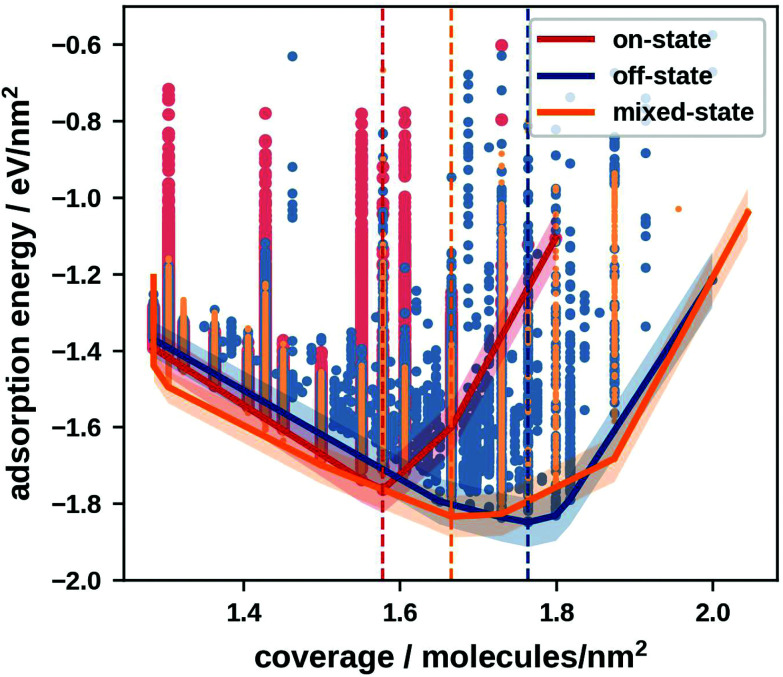
Adsorption energies per area for the 1000 energetically most favourable, as well as the optimised on-, off- and mixed-state motifs at all considered coverages; the lines show the convex hull of the minimum energy; the shaded areas around the convex hulls show the prediction uncertainty.

Fig. 2 shows these reranked (and optimised) energies per area plotted against the coverage for on-, off- and mixed-state motifs. In this chapter, we consider a temperature of 0 K where close-packed adsorbate layers seek to minimise the energy per area, making this the measure of interest.^33^ At temperatures above 0 K, which we discuss in the next chapter, the Gibbs free energy of adsorption, which contains the energy per area as well as temperature-dependent potentials, becomes the relevant measure.^34^

The different classes of motifs have their global minimum energy motif at different coverages. For the off-state the energetically most favourable motifs are higher-order commensurate, strongly indicating a preference of incommensurate layers. This allows off-state motifs to pack more densely than on- and mixed-state motifs. The energetically most favourable off-state motif (and overall energetically most favourable motif) has a coverage of 1.76 molecules nm^−2^. Mixed-state motifs also profit from closer packing. Although they are commensurate, the off-state geometries can adjust their positions relatively freely, allowing for more beneficial molecule–molecule interactions and tighter packing than in on-state motifs. Mixed-state motifs have a minimum at a coverage of 1.67 molecules nm^−2^. On-state motifs have the lowest packing density with the energetic minimum occurring at a coverage of 1.58 molecules nm^−2^. This is due to the strongly corrugated PESs of on-state geometries which prevents them from leaving their potential wells and thus makes it difficult to achieve favourable molecule–molecule interactions (see Fig. 1).

Isolated on-state geometries have an approximately 0.10 eV more attractive molecule–substrate interaction than off-state geometries. However, in a tightly packed layer the molecules exhibit attractive molecule-molecule interactions. These interactions outweigh the energy penalty accrued from off-state geometries. This makes densely packed off- and mixed-state motifs energetically more favourable than on-state motifs.

### 2.3 Switchable interface states

Having discussed possible interface motifs, we will now investigate how to use them for a switchable interface. This requires controllably shifting the work-function change (Δ*Φ*) or the coherent fraction by a measurable, or better, a technically applicable margin. Hereby, the coherent fraction is technologically less relevant, but allows validating the switching behaviour, since it is not directly related to Δ*Φ*. Conversely, Δ*Φ* is highly relevant for devices^35–37^ and will therefore serve as focus of this discussion. For instance, a change in Δ*Φ* of 200 meV would correspond to the typical threshold voltage of a germanium diode. Therefore, the motifs should provide an equally large or larger variety of Δ*Φ*s. To test this, we will present an overview of the possible Δ*Φ*s and coherent fractions.

In off-state motifs the molecules are flat and assume very similar adsorption heights. The geometric uniformity of different possible polymorphs leads to similar Δ*Φ*s with differences being largely due to coverage. Motifs with similar coverage exhibit Δ*Φ*s that vary by only 100 meV and the largest Δ*Φ* is −690 meV. Due to the similar adsorption geometries all off-state motifs exhibit coherent fractions of C-atoms (as well as N- and Cl-atoms) of close to 1.00.

In on-state structures the molecules are strongly bent. Moreover, the different on-state geometries exhibit different adsorption heights, bending and tilting. These dissimilarities result in motifs with similar coverages exhibiting Δ*Φ*s that differ by as much as 600 meV from each other. The maximum Δ*Φ* is −1020 meV. Furthermore, the distortions of the adsorption geometries result in different *z*-positions of the C-atoms, which leads to coherent fractions as low as 0.76. We note in passing that the impact of the internal molecular geometry on the coherent fractions is known and has been used to elucidate adsorption geometries.^29,38^

Mixed-state motifs contain flat lying off-state geometries as well as strongly distorted on-state geometries. Due to different adsorption geometries, mixed-state motifs of similar coverage exhibit Δ*Φ*s that differ by as much as 500 meV from each other. The largest Δ*Φ* is −930 meV. Since molecules sit at different adsorption heights, the *z*-position of C-atoms in off-state geometries is approximately 1.2 Å higher than that in on-state geometries, which is about half of the Pt lattice distance (2.35 Å). Therefore, individual motifs exhibit coherent fractions for C-atoms that are as low as 0.01. Coherent fractions smaller than 1 are sometimes taken to indicate a disordered structure.^39,40^ However, our motifs are highly ordered and commensurate. While thought experiments have shown the possibility of such a behaviour,^29^ this is, to our knowledge, the first report of such low coherent fractions for a system of lying molecules which could, in principle, be observed in an experiment. This effect is seen most strongly for C- and N-atoms and is less pronounced for Cl-atoms. For additional plots and details regarding Δ*Φ* and the coherent fraction see the ESI.†

**Fig. 3:**
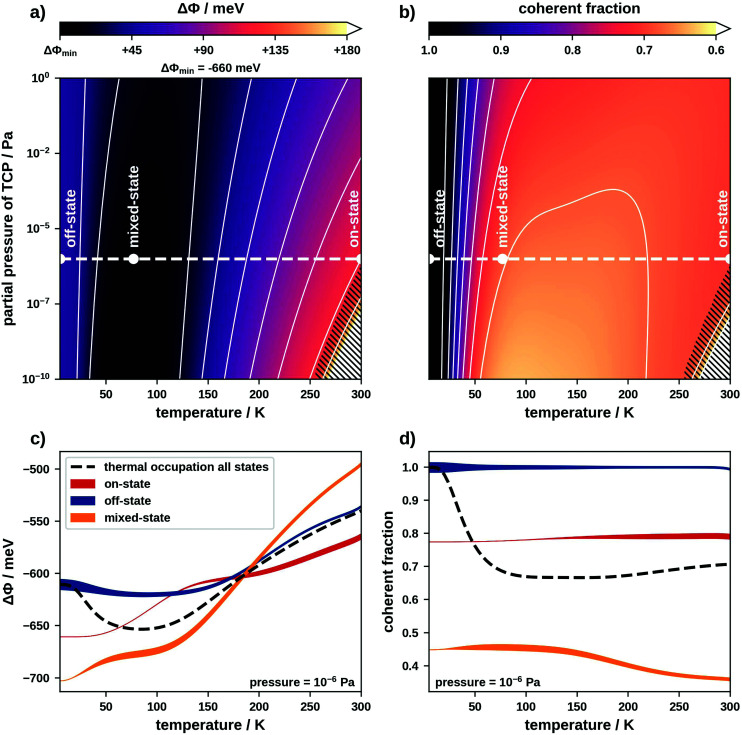
(a and b) Thermodynamically populated phase diagram showing (a) the expectation value of Δ*Φ* and (b) the expectation value of the coherent fraction of C atoms, hatched areas indicate the thermodynamic range where adsorption is not energetically favourable; (c and d) cut through the phase diagram at a constant pressure of 10^−6^ Pa showing expectation values for (c) Δ*Φ* and (d) the coherent fraction separately for on-, off- and mixed-state motifs; the line width shows how much each type of motif contributes to the thermal occupation of all states.

This discussion shows that on-, off- and mixed-state motifs theoretically have a large enough diversity in Δ*Φ*s and coherent fractions to construct a switchable interface. Now we must find a way to control this diversity. An obvious strategy would be using temperature and pressure to shift the thermodynamic equilibrium and thereby influence which motifs form. In thermodynamic equilibrium the thermal occupation governs the probability of finding a particular motif. In fact, multiple motifs may coexist on the surface and contribute to an average Δ*Φ* or coherent fraction. Therefore, we must consider a set of motifs, rather than just the energetically most favourable one. We use a sufficiently large set containing the 37 000 energetically most favourable motifs as well as the clean Pt(111) surface (details in the ESI†). To include the influence of temperature and pressure in the adsorption energies of these motifs we use *ab initio* thermodynamics.^34^ This allows generating the phase diagrams which we show in Fig. 3. Panels (a) and (b) show how the expectation values of Δ*Φ* and the coherent fraction depend on the temperature and the partial pressure of molecules in gas phase. Panels (c) and (d) show the temperature dependence (at a constant pressure of 10^−6^ Pa) of both properties separated into contributions of on-, off- and mixed-state motifs. Regarding Fig. 3c, we note that initially one would expect that the Δ*Φ* of mixed-state motifs lies between that of on- and off-state motifs. However, mixed-state motifs have the lowest expectation values for Δ*Φ* below 200 K and the highest one above. This results from the fact that here the adsorption energies do not correlate with Δ*Φ* and on-state motifs with large Δ*Φ* are not energetically favourable (see ESI†).

Within the phase diagram we define a number of experimentally accessible temperatures and pressures at which we readout the interface state. Pressure in our case refers to the partial pressure of the TCP molecules in gas phase. Since, in practice, reversibly switching the pressure is comparatively difficult, we will primarily switch our interface using temperature. For the discussion we consider temperatures of common coolants, namely that of liquid helium (≈4 K), that of liquid nitrogen (≈77 K) and room temperature (≈300 K).

At 4 K, the thermal occupation is dominated by higher-order commensurate off-state motifs (off-state interface). Here we predict a Δ*Φ* of approximately −600 meV, which matches the contribution from off-state motifs in Fig. 3c. Since molecules in off-state motifs adsorb at similar adsorption heights and are undistorted, the expectation value for the coherent fraction is 1.00 (see Fig. 3d).

At 77 K, mixed-state motifs dominate the thermal occupation (mixed-state interface). Here we find a Δ*Φ* of approximately −650 meV in accordance with the expectation value for mixed-state motifs in Fig. 3c. The fact that these motifs contain both on- and off-state geometries (which adsorb at different heights) leads to a decrease of the coherent fraction to about 0.67.

At 300 K, a large number of motifs, including off- and mixed-state motifs, contribute to the thermal occupation (see ESI†). However, on-state motifs constitute the majority (on-state interface). The Δ*Φ* lies between −550 meV and −500 meV. The coherent fraction at 300 K is approximately 0.70. Furthermore, at partial pressures lower than approximately 10^−7^ Pa it is no longer energetically favourable for molecules to adsorb on the substrate at all (indicated by the hatched areas in Fig. 3).

In passing, we must discuss the uncertainty of our prediction method and the impact this has on the phase diagram. The uncertainty of our adsorption energy predictions is approximately 0.04 eV per molecule. The small energy differences between off- and mixed-state motifs lie within this uncertainty. This mostly affects the off-state interface, where only very few motifs contribute to the thermal occupation. At higher temperatures a larger number of motifs contribute to the thermal occupation averaging out the prediction error. A detailed investigation of the (minor) influence of these errors on our phase diagrams is provided in the ESI.†

Based on our results, two ways of switching the interface exist: a switch from the off-state to the mixed-state interface shifts the coherent fraction by approximately 0.3. Switching from the mixed-state to the on-state interface shifts Δ*Φ* by more than 100 meV. This is in the order of magnitude of the typical threshold voltage of a germanium diode (200 mV), demonstrating the possibility of using TCP on Pt(111) to construct a switchable interface.

We note that this result is comparable to experimentally found work function shifts in other systems: photoisomerisation allows shifting the work function of azobenzene-based SAMs on gold by 70–125 meV.^41^ Photochromic diarylethene derivatives on gold and indium-tin-oxide allow switching the work function by approximately 150 meV (ref. 42) and 250 meV (ref. 43) respectively.

**Fig. 4:**
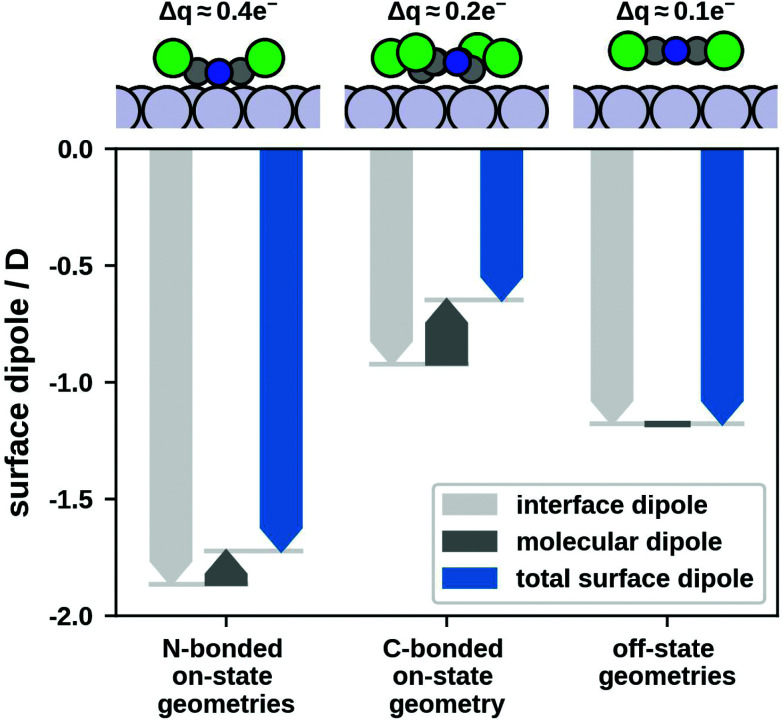
Surface dipoles and (Mulliken) charge transfer for on- and off-state geometries.

However, the amount by which we can switch Δ*Φ* is an order of magnitude smaller than the range of possible Δ*Φ*s. This is partly due to the thermal occupation of energetically higher-lying motifs leading to an averaged interface property. However, the main reason comes to light when looking closely at the adsorption geometries and their surface dipoles. On- and off-state geometries exhibit significantly different adsorption geometries leading to different surface dipoles (see Fig. 4).

Off-state geometries are very similar and have a surface dipole of −1.2 D (data shown for most stable off-state geometry). Since they are physisorbed, interface charge transfer is small with molecules receiving a Mulliken charge of 0.1 electrons.

Conversely, on-state geometries can be separated into two significantly different groups: three on-state geometries bind to the surface *via* N-atoms (N-bonded) while the fourth binds *via* C-atoms (C-bonded). The three N-bonded geometries exhibit a surface dipole of approximately −1.7 D and receive a Mulliken charge of 0.4 electrons (data shown for most stable N-bonded geometry). The C-bonded geometry has a surface dipole of only −0.6 D with a Mulliken charge of 0.2 electrons. However, all on-state geometries have similar adsorption energies. A comparison of the charge transfer and the dipole-induced change of the work function is given in the ESI.†

Therefore, on- and mixed-state motifs containing the C-bonded geometry yield a small Δ*Φ* (comparable to the off-state). Since such motifs are energetically favourable, they contribute to the thermal occupation and decrease the magnitude of the switch in Δ*Φ*. A possible solution would therefore be using a bi-stable system where on- and off-state geometry have similar surface dipoles within the respective state but differ when compared to each other. This could be fulfilled by a molecule that exhibits only one type of bonding chemistry which would lead to more similar on-state geometries.

## 3 Conclusion

In this contribution we discuss a switchable interface using the different adsorption states of TCP on Pt(111). We perform surface structure search and find three different classes of motifs that exhibit different Δ*Φ*s and coherent fractions. Using temperature and pressure as handles we can switch between three interface states. A change from the off-state to the mixed-state interface shifts the coherent fraction by approximately 0.3. More interestingly, switching from the mixed-state to the on-state interface shifts Δ*Φ* by more than 100 meV. This is in the order of magnitude of the typical threshold voltage of a germanium diode (200 mV), demonstrating the possibility of using TCP on Pt(111) to construct a switchable interface.

However, the achieved switch is small compared to the range of possible Δ*Φ*s. Aside from the thermal occupation averaging interface properties, this has two reasons: (i) on-state geometries exhibit different bonding chemistry and surface dipoles and (ii) motifs with extreme Δ*Φ*s are energetically unfavourable. This could be overcome by using a molecule which allows for only one type of bonding chemistry.

## 4 Computational methods

To perform first principles calculations, we use the FHI-aims code^44^ with numerical atom-centered basis functions the PBE exchange-correlation functional,^45^ and the TS^surf^ vdW correction.^46,47^ This approach has proven to yield accurate molecular adsorption geometries when compared to experiments and for metal/organic interfaces, such as PTCDA/Ag(111), has been shown to perform as good as or better than other dispersion-correction-aufmented GGA-type functionals.^48,49^ In this work, we employ well-converged settings as specified in the ESI.† We use periodic boundary conditions to model continuous layers. Since we are dealing with surfaces, we employ the repeated slab approach and decouple the unit cells vertically by using a vacuum of approximately 90 Å as well as a dipole correction.^50^ Furthermore, we use *k*-grids equivalent to a 48 × 48 × 1 grid for the primitive substrate unitcell.

The property we primarily consider is the adsorption energy (or bonding energy) *E*_ads_. We define *E*_ads_ using the total energy of the combined system *E*_mol+sub_, the energy of a tetrachloropyrazine molecule in vacuum *E*_mol_ and the energy of the clean Pt(111) substrate *E*_sub_.

1 *E*_ads_ = *E*_mol+sub_ − *E*_mol_ − *E*_sub_

To perform structure search for commensurate interfaces we use the SAMPLE approach.^24^ SAMPLE uses coarse-graining and Bayesian linear regression. The key premise of this approach is that the unit cell of the adsorbate layer is a supercell of the substrate. This allows us to generate all possible motifs within a given range of coverages and a limited number of molecules per unit cell. The first step of building motifs is generating an exhaustive list of substrate supercells. Then we place molecules into these unit cells. Hereby we coarse-grain the possible positions and orientations of the molecule on the surface. To do so we determine the local minima geometries of the isolated molecule on the substrate. This yields a small number of molecular geometries that we can place in the substrate super cells to assemble motifs. The number of possible motifs we regularly deal with is in the order of 10^6^. Since first principles calculations cannot be done on all these motifs we use an energy model (eqn (2)) to determine the adsorption energies *E*_ads_. The energy model consists of molecule–substrate and molecule–molecule interactions (*U*_i_ and *V*_p_). The sum over all interactions that occur in a motif then yields its energy *E*_ads_.2
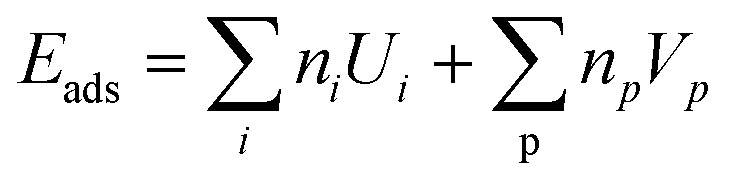


The interactions *U*_*i*_ and *V*_*p*_ are initially unknown. To determine them, we use a type of machine learning called Bayesian linear regression. Hereby, we compute a small, D-optimally selected, number of motifs with DFT. Using these calculations, the Bayesian linear regression algorithm then learns the molecule-substrate and molecule-molecule interactions, which allows predicting the energies of all motifs.

Since we expect that tetrachloropyrazine on Pt(111) forms incommensurate layers, we need the ability to also predict such motifs. Therefore, we generalise the SAMPLE approach (SAMPLE-GPR). We replace the energy model with a Gaussian process, which drops the requirement of discrete molecule-substrate and molecule-molecule interactions. This algorithm is similar to that described in a previous publication.^48^ GPR-based algorithms have been used before to find adsorption geometries of individual molecules on substrates.^51^ Our algorithm can handle isolated molecules on the surface as well as continuous layers. Put simply, a GPR algorithm is a sophisticated method to interpolate adsorption energies and work functions (or other scalar properties). Hereby the key assumption is that two geometries/motifs that are geometrically similar have similar properties. The trick is finding a good measure for this similarity. We use radial distance functions (RDF) *f* which contain interatomic distances between molecules and the substrate as well as interatomic distances between molecules. To determine the similarity *C*_*αβ*_ of two geometries/motifs, *α* and *β*, we only need to calculate the overlap integral between the two RDFs *f*_*α*_ and *f*_*β*_.3
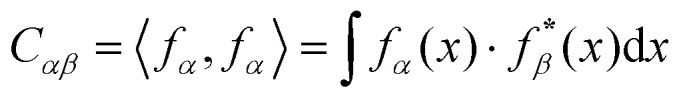


The RDFs are normed such that 〈*f*_*α*_, *f*_*α*_〉 = 1. Therefore, *C*_*αβ*_ is 1 for identical geometries/motifs and yields smaller values for dissimilar geometries/motifs.

Like SAMPLE, SAMPLE-GPR also requires training data. For isolated molecules we evaluate the model uncertainty and choose the data points with the largest uncertainty. In case of continuous adlayers we reuse the training sets from the SAMPLE approach.

To optimise motifs we employ simulated annealing. Hereby SAMPLE-GPR provides the energy predictions. Our algorithm can optimise the most relevant degrees of freedom, namely the unit cell parameters, as well as the positions and orientations of all molecules in the unit cell. In addition to adsorption energies we also use SAMPLE-GPR to learn and predict work functions. However, our model can only handle properties that can be assigned to one molecule, such as energy or dipole. Following the procedure explained and tested in a previous publications [A. Jeindl, L. Hörmann and O.T. Hofmann, Appl. Surf. Sci, 2022, 575, 151687], we convert the work functions into dipoles per molecule. This allows predicting the dipole per molecule for all motifs and converting it back into the work function.

So far we have only discussed surface structure search relying on energies from DFT calculations. These energies do not account for the effects of temperature and pressure, which are vital for a comparison with experiment. To model the impact of different thermodynamic conditions, we use *ab initio* thermodynamics.^34^ Hereby we consider the thermodynamic equilibrium at a given temperature and pressure making the Gibbs free energy of adsorption the measure of interest. When determining the Gibbs free energy we neglect the contributions of the vibration enthalpy, the configuration entropy and the mechanical work as is commonly done in literature.^34,52,53^ Using the Gibbs free energy, we can compute the probability for each motif to occur at a given temperature and pressure. All probabilities combined yield the thermal occupation, which we can use to determine expectation values for Δ*Φ*s and coherent fractions. Therefore, we calculate the mean weighted by the thermal occupation.

For additional details regarding the methods we refer to the ESI.†

## Data availability

The data that support the findings of this study are openly available in the NOMAD repository at https://doi.org/10.17172/NOMAD/2022.03.15-1.

## Conflicts of interest

The authors declare no competing interests.

## Supplementary Material

NR-014-D1NR07763E-s001
