# Supplementary material for: From a bistable adsorbate to a switchable interface: tetrachloropyrazine on Pt(111)
Source: Nanoscale. 2022 Mar 10;14(13):5154–62. doi: 10.1039/d1nr07763e (PMC8972298; doi:10.1039/d1nr07763e)
Supplement: NR-014-D1NR07763E-s001 [file NR-014-D1NR07763E-s001.pdf]

Electronic Supplementary Material (ESI) for Nanoscale.
This journal is © The Royal Society of Chemistry 2022

Electronic Supplementary Information: From a bistable adsorbate to a switchable interface: tetrachloropyrazine on Pt(111)

Lukas Hörmann, Andreas Jeindl, and Oliver T. Hofmann*

Institute of Solid State Physics, Graz University of Technology, Petersgasse 16, 8010 Graz, Austria

E-mail: o.hofmann@tugraz.at

Contents

Convergence Tests **S2**
K-Points S2
Lattice Constant S2
Basis Functions S2
Potential Cutoff S2
Radial Multiplier S2
Gaussian Broadening S3
Substrate Layers Quality S3
Substrate Layers Number S3

Methodological Details **S4**
Predicting Adsorption Energies and Work Function Changes S4
Gas Phase Prior S5
Optimising Motifs S5
Determining the Thermal Occupation S6
Motifs Considered in our Evaluation S6

Results **S7**
Local Adsorption Geometries S7
On-State S7
Off-State S8
Structure Search S9
On-State S9
Off-State S11
Mixed-State S13
Work-Function Change S14
Coherent Fraction S15
Phase Diagrams S15
Probability of Finding On-, Off- and Mixed-State Motifs S16
Comparison of Phase Diagrams S17
Comparison of Phase Diagrams S19

Convergence Tests

K-Points

We perform the k-point convergence using a primitive bulk unit cell of Pt with experimental lattice constant. We use a Γ-centred, equally spaced k-grid, as well as the default "tight" (first tier) species settings of FHI-aims. We find that 48 k-divisions in each lattice direction converge the total energy to within 0.5 meV per atom.

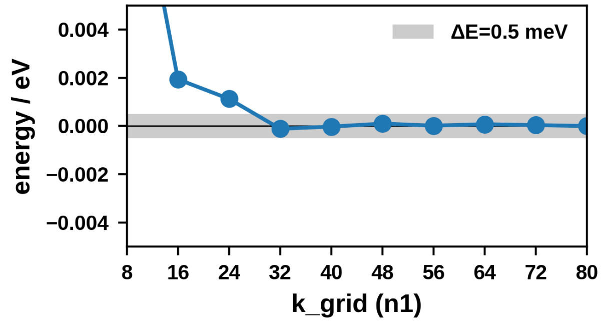

Figure S1: Convergence of a Γ-centred, equally spaced k-grid for a primitive Pt-bulk unit cell

Lattice Constant

We perform the lattice constant convergence using a primitive bulk unit cell with a Γ-centred k-grid with 48 k-points in each direction. The species settings are the default "tight" (first tier) settings of FHI-aims. Using a Birch-Murnaghan fit we find a lattice constant of 2.7755 Å for the conventional unit cell.

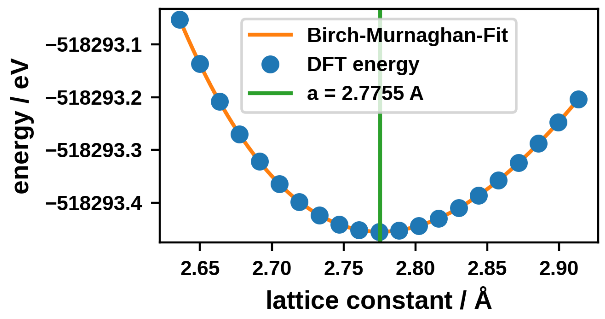

Figure S2: Convergence of the lattice constant of the primitive Pt-bulk unit cell

Basis Functions

We use default "tight" (first tier) basis functions for the upper four substrate layers. For the lower substrate layer we use the minimal basis plus the 4f basis function ("really light" basis set). We use default "tight" (first tier) basis functions for the atom species in the molecule.

Potential Cutoff

We perform the cutoff potential convergence using a 3 × 3 surface unit cell which contains one molecule at an adsorption height of 2.5 Å. The substrate is represented with a mixed-basis slab with 4 layers using the "tight" basis set defaults and 6 layers using the "really light" basis set. The k-grid was chosen to be 12 × 12 × 1. The species settings for the molecule are the default "tight" settings of FHI-aims. We find that a potential cutoff of 4.0 Å converges the adsorption energy per molecule to within 20 meV.

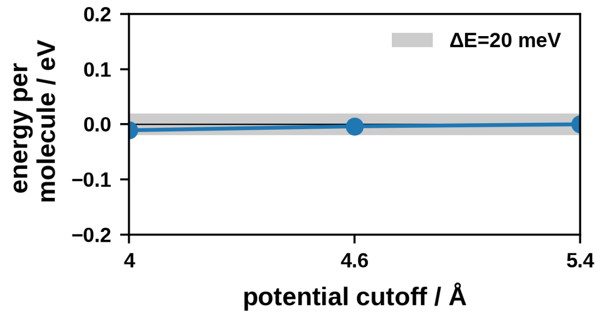

Figure S3: Convergence of the potential cutoff using the adsorption energy of a molecule in a 3 × 3 surface unit cell

Radial Multiplier

We perform the radial multiplier convergence using the same settings as for the potential cutoff. For the potential cutoff we use 4.0 Å. We find that reducing the radial multiplier to 1 for the layers using the "tight" basis set defaults changes the adsorption energy per molecule by only 3 meV. This is within our target accuracy of 20 meV.

Table S1: Convergence of the radial multiplier

radial multiplier	E / eV
2	-57445.1152639
1	-57445.1180491
difference	-0.00278519999

Gaussian Broadening

We perform the convergence of the Gaussian broadening parameter using the same settings as for the potential cutoff and the radial multiplier. For the potential cutoff we use 4.0 Å and for the layers using the "tight" basis set defaults we use a radial multiplier of 1. We find that changing the Gaussian broadening from 0.01 eV to 0.1 eV influences the adsorption energy per molecule by less than 20 meV. For our production calculation we use a Gaussian broadening of 0.01.

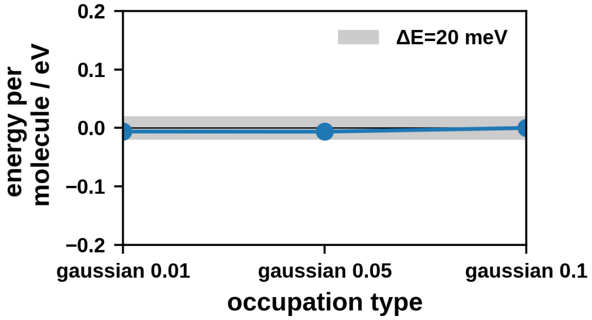

Figure S4: Convergence of the Gaussian broadening parameter using the adsorption energy of a molecule in a 3 × 3 surface unit cell

Substrate Layers Quality

We perform the convergence of the substrate layer quality using a 3 × 3 surface unit cell, which contains one molecule at an adsorption height of 2.5 Å. The k-grid was chosen to be 12 × 12 × 1. The species settings for the molecule are the default "tight" settings of FHI-aims. For the substrate, we start with 10 layers with a "really light" basis set and incrementally replace layers using the "tight" basis set defaults (radial multiplier 1), starting from the top. The adsorption energy converges with 4 layers using the "tight" basis set.

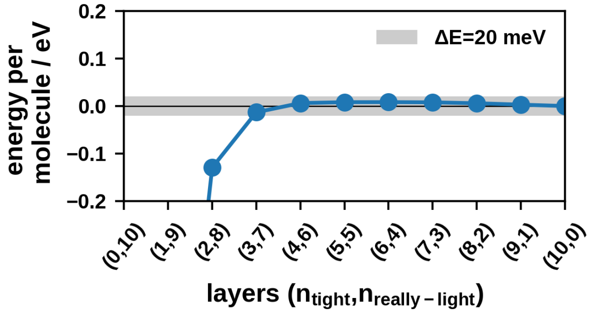

Figure S5: Convergence of required number of layers with the "tight" basis set defaults using the adsorption energy of a molecule in a 3 × 3 surface unit cell

Substrate Layers Number

We perform the convergence of the number of substrate layers using a 3 × 3 surface unit cell containing one molecule 2.5 Å above the substrate. The k-grid was chosen to be 12 × 12 × 1. The species settings for the molecule are the default "tight" settings of FHI-aims. We start with 4 layers using the "tight" basis set defaults and 2 layers using the "really light" basis set. Then we incrementally add layers using the "really light" basis set at the bottom. The adsorption energy converges for 4 layers using the "tight" basis set defaults 3 layers using the "really light" basis set. Out of an abundance of caution we finally use a slab consisting of 4 layers using the "tight" basis set defaults and 4 layers using the "really light" basis set.

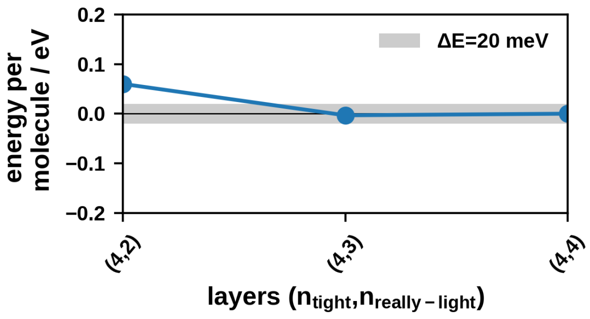

Figure S6: Convergence of required number of layers with the “really light” basis set using the adsorption energy of a molecule in a 3×3 surface unit cell

Methodological Details

Predicting Adsorption Energies and Work Function Changes

To determine adsorption energies and work functions changes ($\Delta\Phi$) for all geometries (individual molecules adsorbed on the substrate) and motifs (adlayers) we use Gaussian process regression (GPR). Our algorithm is similar to that described in a previous publication.[1] Put simply, a GPR algorithm is a sophisticated method to interpolate adsorption energies, $\Delta\Phi$s and other scalar properties. Hence, we have two sets of data points: The first set has properties $\boldsymbol{E_P}$ we want to predict and the second set contains calculated properties $\boldsymbol{E_C}$. Hereby a data point can be a geometry, i.e. an individual molecule on the surface or a motif, i.e. a molecular adlayer. As stated above, in this work the property is either the adsoption energy or the surface dipole, which is directly associated with $\Delta\Phi$. We assume that these properties are connected via a multivariate normal distribution (see equation 1).

$$\begin{pmatrix}\boldsymbol{E_P}\\ \boldsymbol{E_C}\end{pmatrix} \sim \mathcal{N}\left(\begin{pmatrix}\boldsymbol{\mu_P}\\ \boldsymbol{\mu_C}\end{pmatrix}, \begin{pmatrix}C^{PP} & C^{PC}\\ C^{CP} & C^{CC}+\sigma^2\mathbb{1}\end{pmatrix}\right) \tag{1}$$

$\boldsymbol{\mu_C}$ and $\boldsymbol{\mu_P}$ are prior values for the calculated properties and the properties we want to predict respectively. C^{PP}, C^{PC}, C^{CP} and C^{CC} belong to the covariance matrix and σ accounts for the uncertainty of our calculated properties. The normal distribution given in equation 1 can be rewritten into a conditional distribution for $\boldsymbol{E_P}$. This allows calculating the expectation value $\bar{\boldsymbol{\mu}}$ for the set of data points we want to predict. Hereby the algorithm requires the input of calculated properties $\boldsymbol{E_C}$ for a second set of data points (see equation 2).

$$\bar{\boldsymbol{\mu}} = \boldsymbol{\mu_P} + C^{PC}(C^{CC}+\sigma^2\mathbb{1})^{-1}(\mathbf{E_C}-\boldsymbol{\mu_C}) \tag{2}$$

The key ingredient of GPR is arguably the covariance matrix C. It, as stated above, comprises four parts:

- C^{PP} is the covariance matrix between data points we predict.
- C^{PC} is the covariance matrix between data points we predict and points we calculate.
- $(C^{PC})^T = C^{CP}$.
- C^{CC} is the covariance matrix between data points we calculate.

Each single element $C_{\alpha\beta}$ in the covariance matrix can be understood as a measure of similarity between two data points denoted with indices α and β. This similarity must be correlated with the property of the respective data points. Two data points with a large similarity must also have similar energies. We realise this by defining the similarity as the difference between two radial distance functions (RDF). A RDF $\boldsymbol{f_\alpha}$ is sum of Gaussians with width τ, where the mean value of each Gaussians is a distance between two atoms in the data points (see equation 3).

$$\boldsymbol{f_\alpha}(x) = \sum_{i=0}^{N} \frac{1}{\tau\sqrt{2\pi}} \exp\left[-\frac{\left(\left(\frac{d_{\alpha,i}}{d_{min}}\right)^n - x\right)^2}{2\tau^2}\right] \quad (3)$$

Hereby, τ is a hyperparameter and $d_{\alpha,i}$ is the distance between two atoms as depicted by the arrows in Figure S7. N is the total number of possible distances, d_{min} is the minimum distance that atoms can assume within the search space of the GPR algorithm and n is a decay power inspired by Coulomb interactions (if $n = -2$). Each RDF belongs to a data point and can consist of two parts (see Figure S7): The first RFD comprises distances between atoms of the molecule and atoms of the substrate and takes care of the molecule-substrate interactions. This part is required for treating isolated molecules as well as continuous adlayers. The second RDF contains distances between atoms of the molecules. It accounts for the molecule-molecule interactions and is only required when looking at continuous layers of molecules.

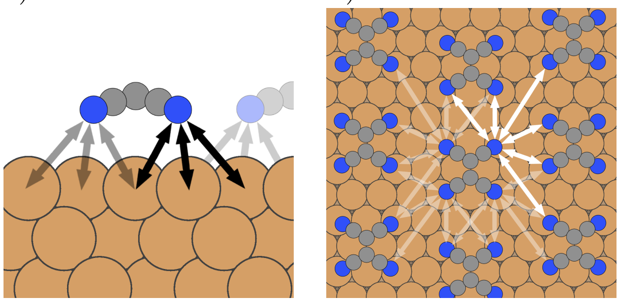

Figure S7: Parts of the RDF: a) RDF for molecule-substrate interactions, b) RDF for molecule-molecule interactions, light and solid colouring only for visibility

To determine the similarity $C_{\alpha\beta}$ of two data points, α and β, we only need to calculate the overlap integral between the two RDFs $\boldsymbol{f_\alpha}$ and $\boldsymbol{f_\beta}$. The RDFs are normed such that $\langle \boldsymbol{f_\alpha}, \boldsymbol{f_\alpha} \rangle = 1$.

$$C_{\alpha\beta} = \langle \boldsymbol{f_\alpha}, \boldsymbol{f_\alpha} \rangle = \int \boldsymbol{f_\alpha}(x) \cdot \boldsymbol{f_\beta^*}(x)\, \mathrm{d}x \quad (4)$$

In principle the GPR algorithm can consider all degrees of freedom. In practice this would make predictions inefficient. Therefore, we only use the most important degrees of freedom. For single molecules on the surface, these are the position, orientation (rotation around the axis perpendicular to the surface) and bending (the softest vibration mode of the molecule in vacuum) of the molecule. For continuous layers we only use position and orientation of the molecule.

Gas Phase Prior

To improve the predictions of SAMPLE and the generalised, GPR based, variant SAMPLE-GPR it is possible to use a gas phase prior. Hereby we train an initial energy model (gas phase model) using DFT calculations of freestanding molecular layers in vacuum. Since these calculations do not contain a metal substrate, they are much less computationally expensive, allowing for training sets with thousands of motifs. Therefore, we can learn molecule-molecule interactions with high accuracy. Provided that the interaction with the substrate (charge transfer, etc.) does not significantly alter the molecule-molecule interactions, the predictions from the gas phase model can be used as prior information when training with molecular layers on the substrate.

Optimising Motifs

To optimise motifs we use simulated annealing. Hereby the GPR algorithm provides the energy predictions. The simulated annealing algorithm works as follows:

First, the algorithm starts with a commensurate motif.

Second, the algorithm selects a motif in the neighborhood of the initial motif by randomly choosing new unit cell parameters and scaling the positions of the molecules to fit the new

unit cell. Hereby we restrict the possible unit cell parameters to guarantee that the number of molecules in the newly generated higher-order commensurate motif remains below a given threshold.

Third, the algorithm performs a BFGS optimisation of the positions and orientations of all molecules in the unit cell. This allows finding local minima for higher-order commensurate motifs similar to the motifs determined by the SAMPLE approach. A step is accepted according to the probability p_A given by the Boltzmann statistics (see equation 5), where E_{best} is the best energy found so far, E_n is the energy of the current step, and β is an inverse temperature. The inverse temperature increases in each iteration.

$$p_A = \min\{1, \exp(-\beta \cdot (E_n - E_{best}))\} \quad (5)$$

Steps two and three are repeated until the convergence criterion is reached. We consider an optimisation as converged if no better motifs have been found for more than 20 iterations.

Determining the Thermal Occupation

So far we have discussed finding possible motifs as well as their adsorption energies. However, these energies are only valid for 0 K. If we consider the thermodynamic equilibrium at a given temperature and pressure the measure of interest is the Gibbs free energy of adsorption $\gamma_\alpha(p, T)$, for which we use ab-intio thermodynamics.[2] We neglect the contributions of the vibration enthalpy, the configuration entropy and the mechanical work as is commonly done in literature.[2–4] This yields equation (6).

$$\gamma_\alpha(p, T) = E_\alpha - \frac{1}{A_\alpha} \cdot \mu(p, T) \quad (6)$$

Here, E_α is the energy per area and A_α is the area per molecule of motif α. $\mu(p, T)$ is the chemical potential of the TCP (tetrachloropyrazine) molecules in gas phase. The Gibbs free energy of adsorption allows determining the probability to find a particular motif in an experiment at a given temperature and pressure. We determine the probability $p_\alpha(T, p)$ for each motif α to occur by calculating the Boltzmann distribution.

$$p_\alpha(p, T) = \frac{1}{Z} \exp\left(-\frac{\gamma_\alpha(p, T)}{k_B T} \cdot \bar{A}\right) \quad (7)$$

Here γ_α is the Gibbs free energy of adsorption of motif α. $\bar{A}$ is a reference area which we self-consistently determine via equations (7) and (8). A more thorough explanation is given by Jeindl et al..[5]

$$\bar{A} = \frac{1}{\sum_\alpha p_\alpha / A_\alpha} \quad (8)$$

The probabilities $p_\alpha(T, p)$ from the Boltzmann distribution can be interpreted as the relative area a particular motif occupies on the surface. This allows determining the expectation value of different interface properties such as $\Delta\Phi$ or the coherent fraction, at different temperatures and pressures. Here y_α is the property of motif α.

$$\overline{y}(p, T) = \sum_\alpha p_\alpha(p, T) \cdot y_\alpha \quad (9)$$

Motifs Considered in Our Evaluation

In total, we determine approximately 3 million motifs. For our evaluations we use a subset of motifs, which is sufficiently large to guarantee that all our results are converged. This subset contains approximately 37000 motifs. We select these motifs in the following way:

- For each class of motif and each coverage we include the 1000 most energetically favourable motifs.
- For off-state motifs, we optimise the 20 motifs with the lowest energy per area

with simulated annealing. We include all accepted steps of the simulated annealing run.

- For mixed-state motifs, we optimise the 40 most energetically favourable motifs of every coverage and include the optimised motifs.

To test if our subset is large enough we determine how many motifs contribute to the thermal occupation at different temperatures. This is shown in Figure S8. Panel (a) shows the weight (given by the Boltzmann distribution) with which a particular motif influences the thermal occupation plotted over the Gibbs free energy of adsorption γ relative to its minimum γ_{min}. For low temperatures the most favourable motif has a weight of close to 1 and the contribution of less favourable motif decays quickly. For larger temperatures the individual contributions of even the most favourable motifs are only in the order of 10^{-3}. Hence, a large number of motifs contributes to the thermal occupation. Panel (b) shows the cumulative probability distribution. For small temperatures only motifs within a window of $\gamma - \gamma_{min} \approx 0.01\ eV$ contribute to the thermal occupation. For larger temperatures this window increase to $\gamma - \gamma_{min} \approx 0.15\ eV$. Panel (c) shows a histogram of the number of motifs at different values of $\gamma - \gamma_{min}$. The dashed line indicates the number of motifs making up 99 % of the contribution of the thermal occupation at 300 K. This shows that approximately 23000 motifs contribute to the thermal occupation at room temperature. Further, 14000 configurations do not contribute significantly to the thermal occupation demonstrating that the set of 37000 motifs is sufficiently large.

Results

Local Adsorption Geometries

On-State

To find the local adsorption geometries (or geometries for short) for the on-state we perform two steps. First, we do a rough pre-search using approximations and cheaper computational settings (see below). Second, we use the geometry candidates found in the first step as starting points for DFT geometry optimisations where we use converged settings.

To determine the approximate PES of the single molecule on the surface, we use our GPR algorithm with five degrees of freedom. These are the three spatial coordinates of the centre of mass of the molecule, the rotation around the axis perpendicular to the substrate, as well as the softest vibration mode of the molecule in vacuum. These degrees of freedom allow us to determine a good approximation of the PES. We use a two layer substrate and otherwise the same convergence settings described above. With these setting we calculate 80 training points where the molecule assumes different positions, orientations and bending on the substrate. This allows us to interpolate the PES and to determine all minima (i.e. geometry candidates), which are displayed in Figure S9.

The approximate PES is strongly corrugated which reflects on the optimal adsorption height at different lateral positions of the molecule on the surface (see Figure S10). In Figure S10 the molecule is oriented as shown in the figure while the bending is optimised at every position. We find substantial differences in the optimal adsorption height at different lateral positions.

As stated above, we use the geometry candidates from the first step as starting points of DFT geometry optimisations. Hereby we relax the molecule as well as the first two substrate layers. This is necessary, since the substrate relaxation contributes a large gain in bonding energy. The computational settings correspond to the converged values presented above and the convergence criterion for the remaining force is 0.01 $eV/Å$. The eleven starting points converge to six different geometries depicted in Figure S11. We use the four most energetically favourable of these as the on-state geometries to determine motifs.

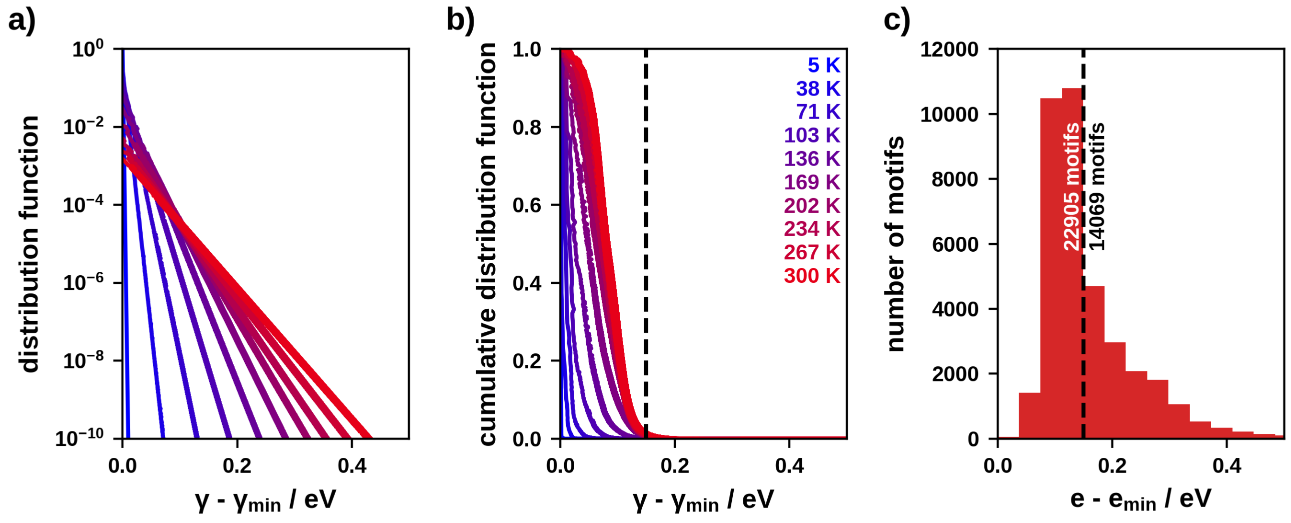

Figure S8: Visualisation of the (a) contribution of each structure to the thermal occupation (Boltzmann distribution), (b) the cumulative distribution and (c) the number of motifs contributing to the thermal occupation at different temperatures

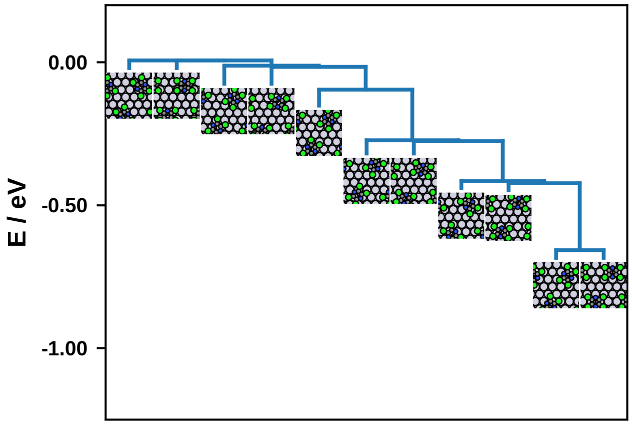

Figure S9: Minima and barriers for the approximate on-state PES

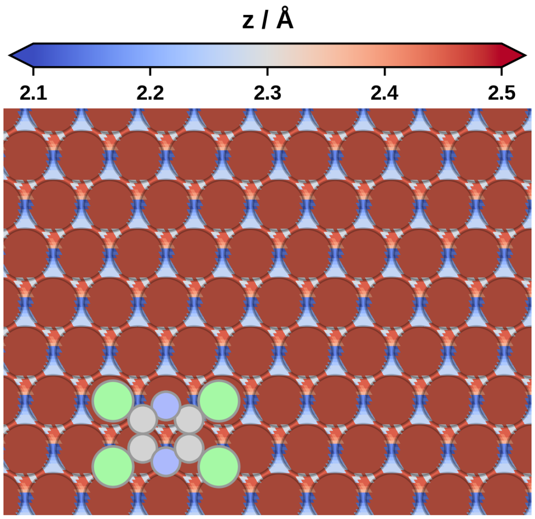

Figure S10: Optimal adsorption height at different lateral positions of the on-state geometry

Off-State

Compared to the on-state, the off-state PES is much less complex. This is due the fact that in the off-state the molecules mainly bond to the surface via van der Waals interactions, which are comparatively uniform. Hence, GPR alone is sufficient to perform the search for off-state geometries. To determine the PES, we use four degrees of freedom. These are the three spatial coordinates of the centre of mass of the molecule, the rotation around the axis perpendicular to the substrate. In the off-state the molecule does not bend so a fifth degree of freedom is not necessary. Use the convergence settings described above, we calculate 50 training points where the molecule assumes a different position and orientation on the substrate. These training data allows interpolating the PES, which we then probe for all minima (i.e. geometry candidates),

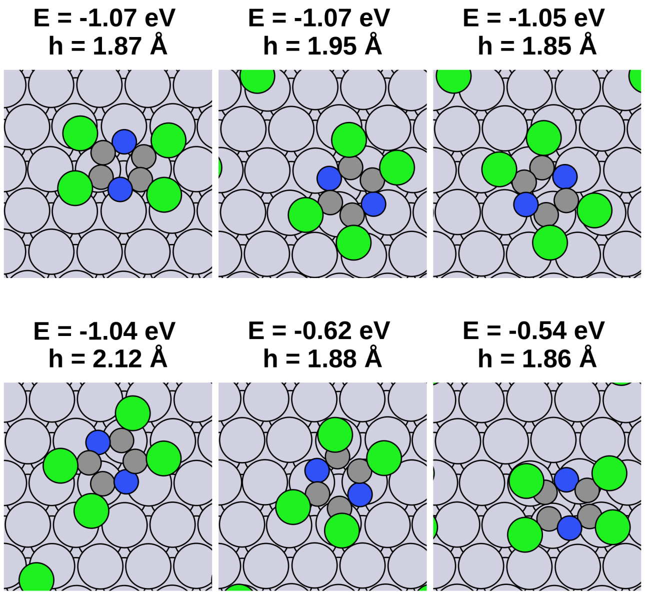

Figure S11: Geometry-optimised on-state geometries

which are displayed in Figure S12. We use all of these as the off-state geometries to determine motifs. Additional DFT geometry optimisations are not necessary, since (i) the energy gain is within the uncertainty of our prediction methods and (ii) we later optimise the positions and orientations of molecules in off-state motifs.

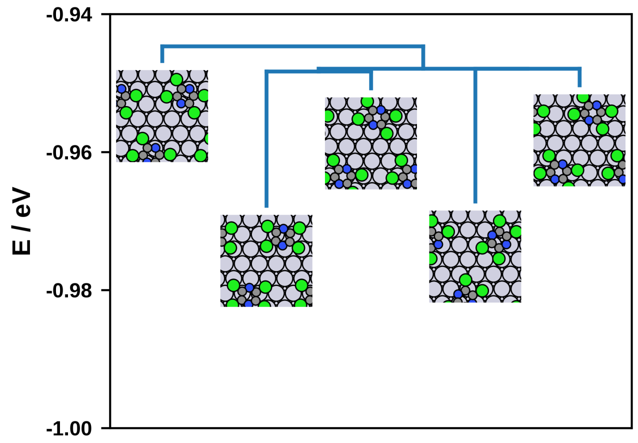

Figure S12: Minima and barriers for the off-state PES

Figure S13 shows the optimal adsorption height at different lateral positions of the molecule on the surface. Hereby the molecules is oriented as show in the Figure. We find only small differences in the optimal adsorption height at different lateral positions.

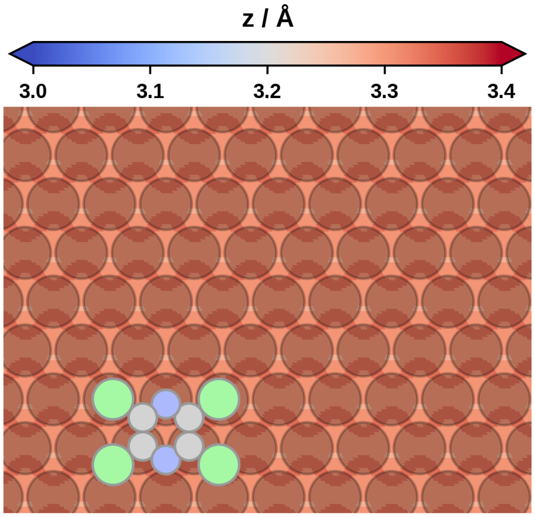

Figure S13: Optimal adsorption height at different lateral positions of the off-state geometry

Structure Search

We perform structure search in three steps. First, we determine the energies of all commensurate motifs with SAMPLE. Second, we rerank the best 1000 motifs of each coverage with SAMPLE-GPR. Third, we optimise the off- and mixed-state motifs. For the off-state motifs we optimise all adsorbates as well as the coverage. In case of the mixed-state motifs, we optimise the position and orientation of the off-state geometries.

On-State

SAMPLE convergence There exist a number of hyperparameters in SAMPLE. For most of these one can find physically or numerically motivated settings. However, two parameters, namely the feature correlation length and the decay length, need to be optimised. We optimise these hyperparameters by minimising the prediction error on a test set of gas phase calculations. The training set contains 200 D-optimally selected gas phase

calculations and the test set comprises 3000 calculations (which includes the training data).

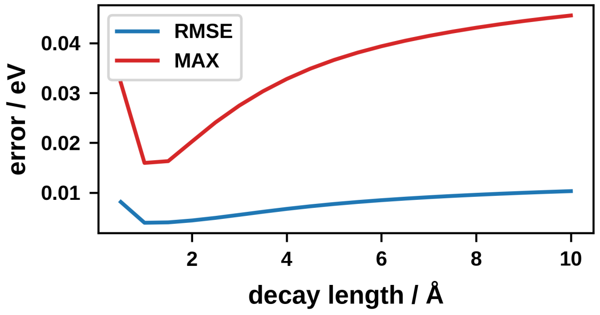

Figure S14: Optimisation of the decay length

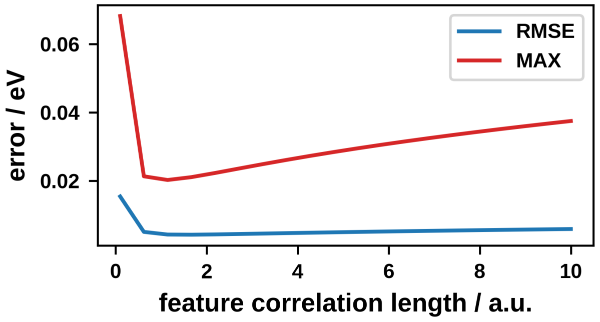

Figure S15: Optimisation of the feature correlation length

Figures S14 and S15 show that a feature correlation length of 1 and a decay length of 1 Å yield the minimum test set error. Table S2 shows the settings for all hyperparameters.

Table S2: On-state hyperparameters used in the SAMPLE approach

distance cutoff	16 Å
distance threshold Cl-Cl	2.7 Å
distance threshold Cl-N	2.5 Å
distance threshold N-N	2.4 Å
feature threshold	0.01
feature correlation length	1
decay length	1 Å
decay power	-2
one-body std	100 meV
two-body std	100 meV
DFT noise	5 meV
feature dimension Cl-Cl	16
feature dimension Cl-N	8
feature dimension N-N	4

Figure S16 shows the learning curve using gas phase calculations. The error is determined on a set of 3000 calculations that also include the training set.

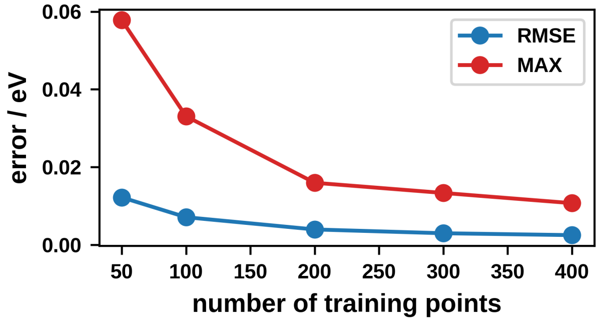

Figure S16: Learning curve for free-standing molecular layers

SAMPLE Predictions To train the energy model for motifs on the substrate we d-optimally select 100 on-substrate calculations. Figure S17 shows the predicted energies for all commensurate on-state motifs with up to three molecules per unit cell. We find a RMSE of 0.03 $eVnm^{-2}$.

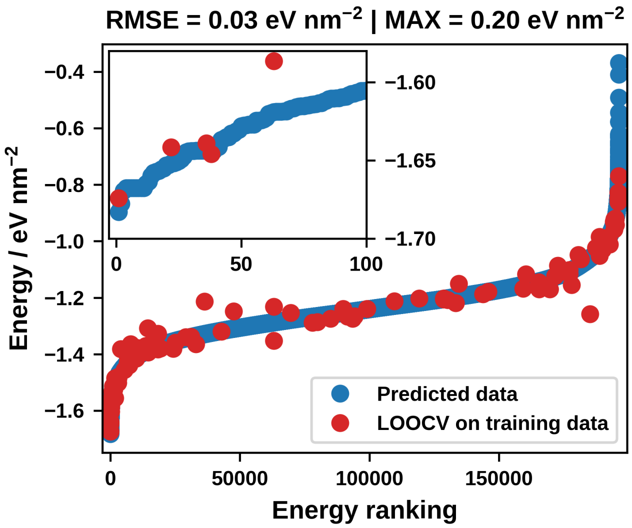

Figure S17: SAMPLE energy ranking and leave one out cross validation of calculated energies

SAMPLE-GPR To train SAMPLE-GPR we reuse the 100 on-substrate calculations from the SAMPLE training set. Figure S18 shows the learning curve. The hyperparameters are optimised by maximising the log marginal likelihood. We find a RMSE of 0.039 $eV/molecule$.

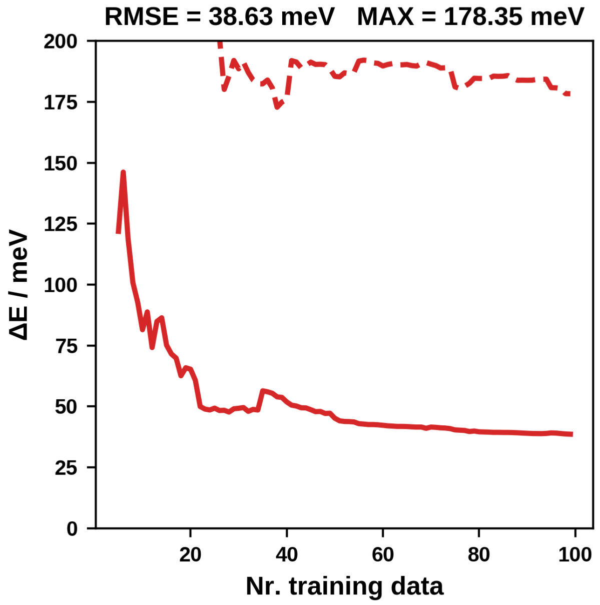

Figure S18: GPR learning curve with leave one out cross validation error

Off-State

SAMPLE Convergence We optimise two hyperparameters, namely the feature correlation length and the decay length by minimising the prediction error on a test set of gas phase calculations. The training set contains 200 D-optimally selected gas phase calculations and the test set comprises 3000 calculations of commensurate motifs (which includes the training data).

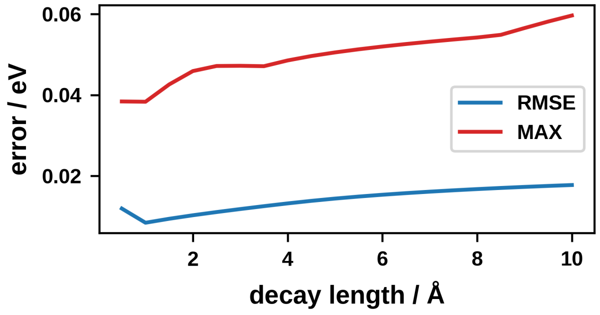

Figure S19: Optimisation of the decay length

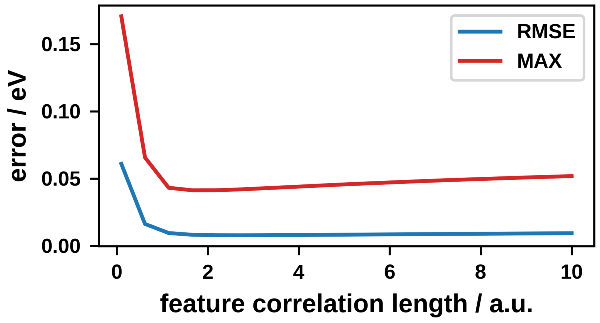

Figure S20: Optimisation of the feature correlation length

Figures S19 and S20 show that a feature correlation lenght of 1 and a decay length of 1 Å yield the minimum test set error. Table S3 shows the settings for all hyperparameters.

Table S3: Off-state hyperparameters used in the SAMPLE approach

distance cutoff	16 Å
distance threshold Cl-Cl	2.7 Å
distance threshold Cl-N	2.5 Å
distance threshold N-N	2.4 Å
feature threshold	0.01
feature correlation length	1
decal length	1 Å
decay power	−2
one-body std	100 meV
two-body std	100 meV
DFT noise	5 meV
feature dimension Cl-Cl	16
feature dimension Cl-N	8
feature dimension N-N	4

Figure S21 shows the learning curve using gas phase calculations. The error is determined on a set of 3000 calculations that also includes the training set.

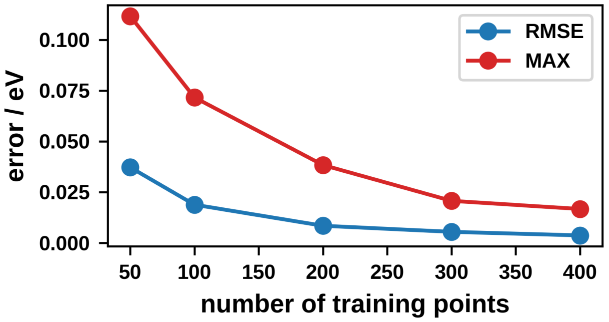

Figure S21: Learning curve for free-standing molecular layers

SAMPLE Predictions To train the energy model for motifs on the substrate we d-optimally select 50 on-substrate calculations. Additionally, we use a gas phase prior which employs a training set of 3000 gas phase calculations. Figure S22 shows the predicted energies for all commensurate off-state motifs with up to three molecules per unit cell. The RMSE is 0.02 $eV nm^{-2}$.

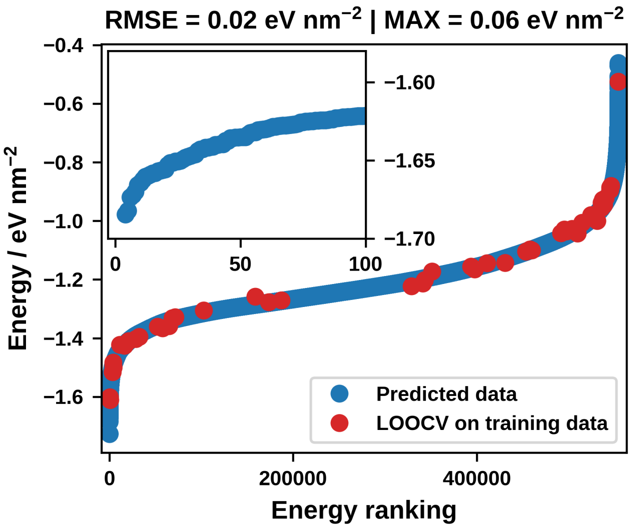

Figure S22: SAMPLE energy ranking and leave one out cross validation of calculated energies

SAMPLE-GPR To train SAMPLE-GPR we reuse the 50 on-substrate calculations from the SAMPLE training set. For the gas phase prior we use a new training set comprising 2263 commensurate and higher-order commensurate motifs. Figure S23 shows the learning curve. The learning curve shows no improvement of LOOCV error when adding new data points. This likely results from the fact that most information of the molecule-molecule interactions is already being contained in the gas phase prior. The small RMSE of 0.037 $eV/molecule$ fits to this assertion. We note in passing that the hyperparameters are optimised by maximising the log marginal likelihood.

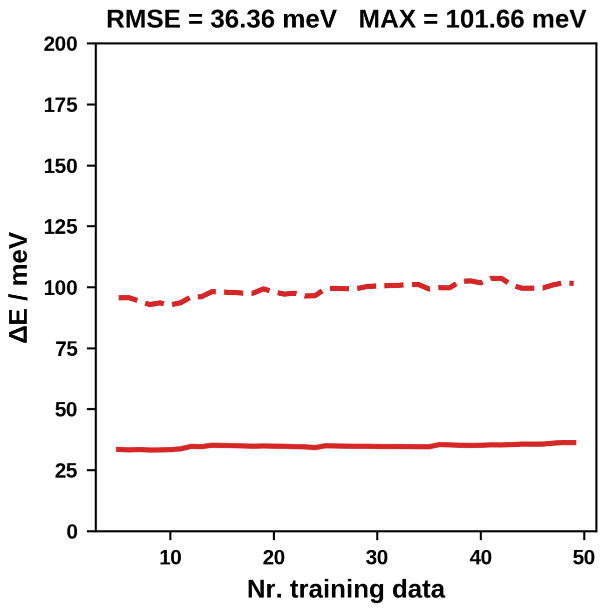

Figure S23: GPR learning curve with leave one out cross validation error

Mixed-State

SAMPLE Convergence We optimise the feature correlation length and the decay length by minimising the prediction error on a test set of gas phase calculations. The training set contains 200 D-optimally selected gas phase calculations and the test set comprises 3000.

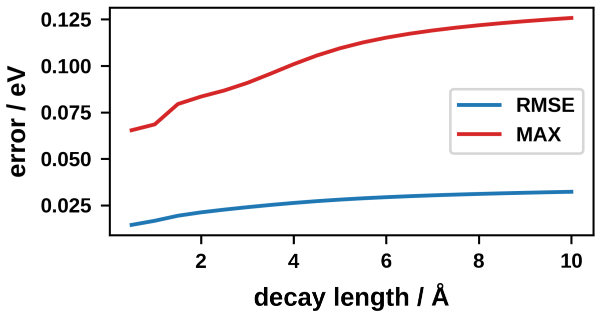

Figure S24: Optimisation of the decay length

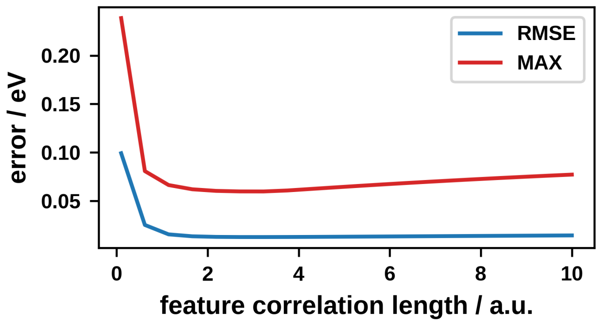

Figure S25: Optimisation of the feature correlation length

Figures S24 and S25 show that a feature correlation length of 1 yield the minimum test set error. Regarding the decay length, we find that smaller values would further reduce the test set error, albeit by only a small amount. To be consistent with the on- and off-state we use a decay length of 1 Å. Table S4 shows the settings for all hyperparameters.

Table S4: Mixed-state hyperparameters in SAMPLE

distance cutoff	16 Å
distance threshold Cl-Cl	2.7 Å
distance threshold Cl-N	2.5 Å
distance threshold N-N	2.4 Å
feature threshold	0.01
feature correlation length	1
decal length	1 Å
decay power	-2
one-body std	100 meV
two-body std	100 meV
DFT noise	5 meV
feature dimension Cl-Cl	16
feature dimension Cl-N	8
feature dimension N-N	4

Figure S26 shows the learning curve using gas phase calculations. The error is determined on a set of 3621 calculations that also include the training set. We determine a RMSE of 0.04 $eV nm^{-2}$.

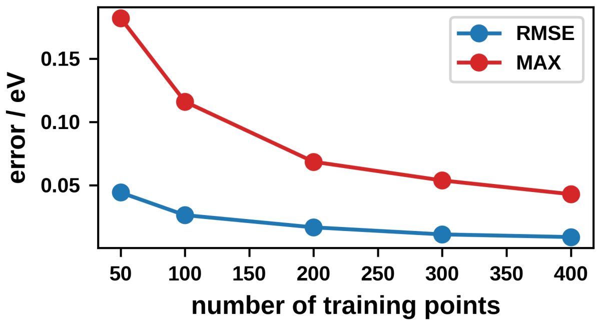

Figure S26: Learning curve for free-standing molecular layers

SAMPLE Predictions To train the energy model for motifs on the substrate we d-optimally select 75 on-substrate calculations. Additionally, we use a gas phase prior which employs a training set of 3621 gas phase calculations. Figure S27 shows the predicted energies for all commensurate mixed-state motifs with up to three molecules per unit cell.

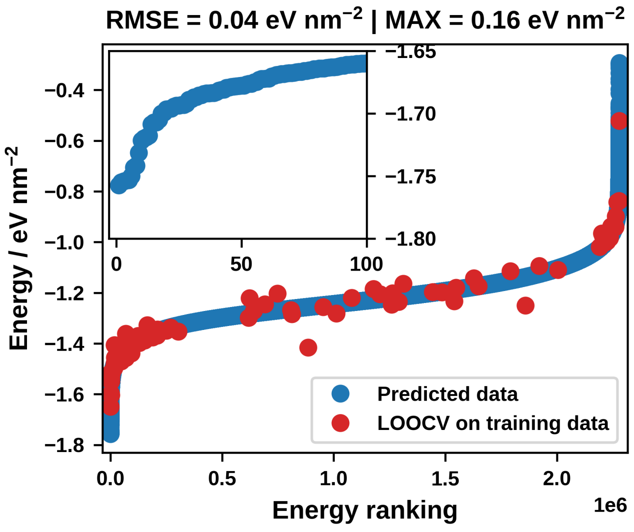

Figure S27: SAMPLE energy ranking and leave one out cross validation of calculated energies

SAMPLE-GPR We train SAMPLE-GPR by reusing the 75 on-substrate calculations from the SAMPLE training set. For the gas phase prior we use a new training set comprising 3621 motifs where in some instances the off-state molecules were slightly shifted. Figure S28 shows the learning curve. The RMSE amounts to 0.033 $eV/molecule$ is comparable to SAMPLE. The hyperparameters are optimised by maximising the log marginal likelihood.

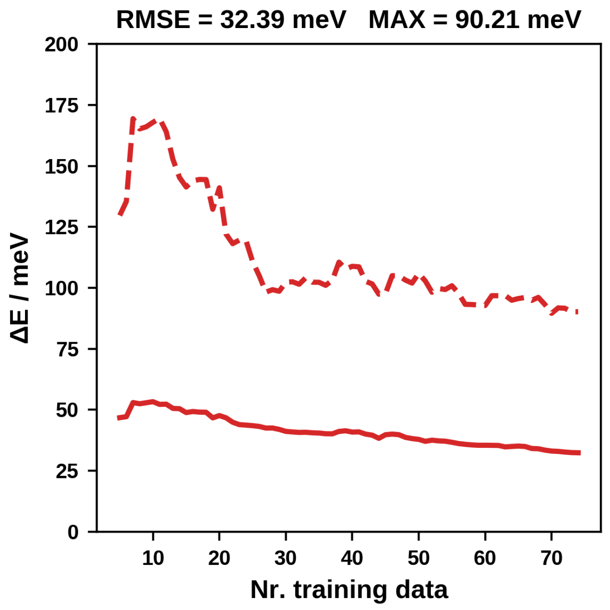

Figure S28: GPR learning curve with leave one out cross validation error

Work-Function Change

Figure S29 shows the adsorption energies plotted against the $\Delta\Phi$ for the subset containing 37000 motifs. We find that on-state motifs exhibit the largest variety of $\Delta\Phi$s. Within our range of coverage the largest $\Delta\Phi$ is -1020 meV and the smallest is -330 meV. This is due to dissimilar absorption geometries, which have significantly different surface dipoles. For the same reason, mixed-state motifs show the second largest variety of $\Delta\Phi$s. Here the largest $\Delta\Phi$ is -932 meV and the smallest is -307 meV, within our range in coverage. Conversely, off-state motifs contain only geometrically very similar adsorption geometries, which leads to a small variety of $\Delta\Phi$s. We find the largest $\Delta\Phi$ to be -688 meV and the smallest is -444 meV. In this case the variety is mainly driven by the coverage.

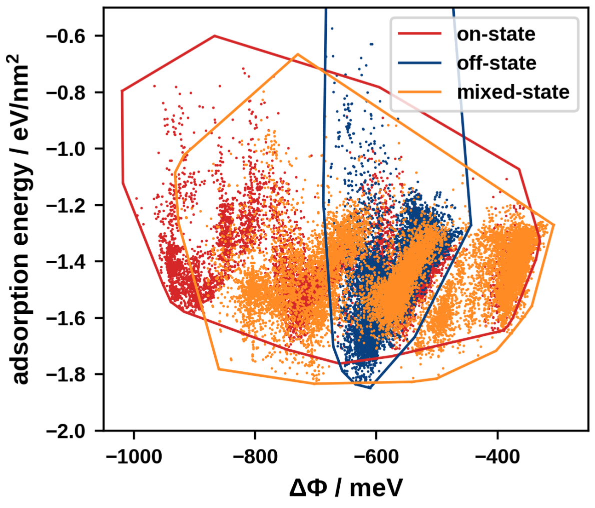

Figure S29: Adsorption energies plotted against the $\Delta\Phi$

Coherent Fraction

Figure S30 shows the adsorption energies plotted against the coherent fraction for the subset containing 37000 motifs.

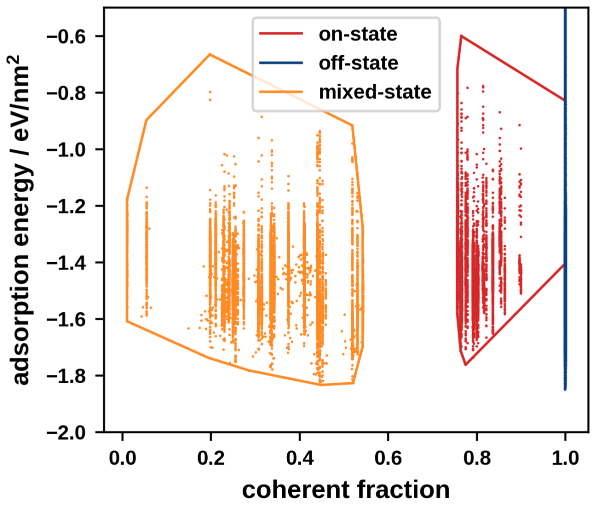

Figure S30: Adsorption energies plotted against the coherent fraction for C-atoms

Off-state motifs have the largest coherent fraction with all structure exhibiting a value of close to 1.00. This is due to the molecules remaining flat and adsorbing at similar heights. On-state motifs exhibit coherent fractions ranging from approximately 0.75 to 1.00. Although all molecule in this case adsorb at similar heights, they can have different adsorption geometries, leading to lower coherent fractions. Mixed-state motifs exhibit the lowest coherent fraction with values ranging from approximately 0.00 to 0.55. The reason for this is that the mixed-state contains molecules adsorbed at different heights.

Phase Diagrams

Figures S31, S32 and S33 show the phase diagrams of the expectation values for the coherent fractions of C-, Cl- and N-atoms respectively. We find the largest differences in coherent fraction for C-atoms. The smallest differences occur for Cl-atoms. This is due to the fact that the Cl-atoms are bent upwards in on-state geometries and therefore lie at approximately the same height as the Cl-atoms in the flat off-state geometries.

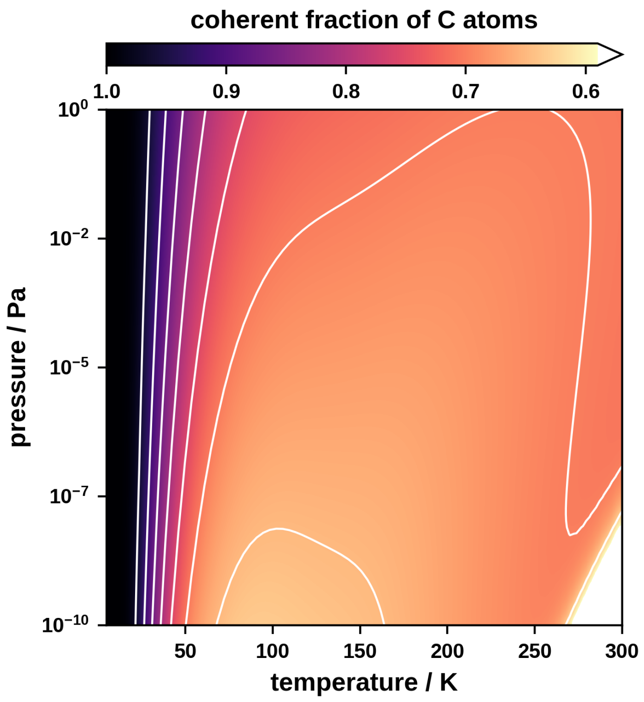

Figure S31: Thermodynamically populated phase diagram showing the expectation value of the coherent fraction for C atoms

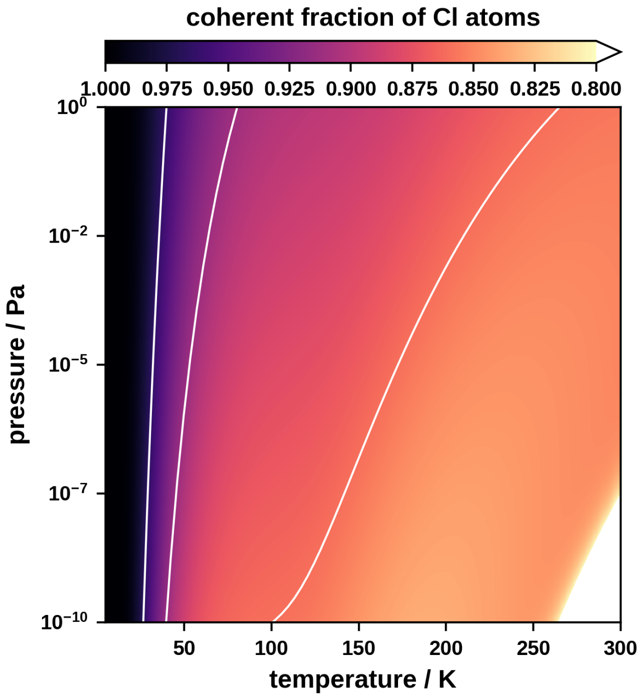

Figure S32: Thermodynamically populated phase diagram showing the expectation value of the coherent fraction for Cl atoms

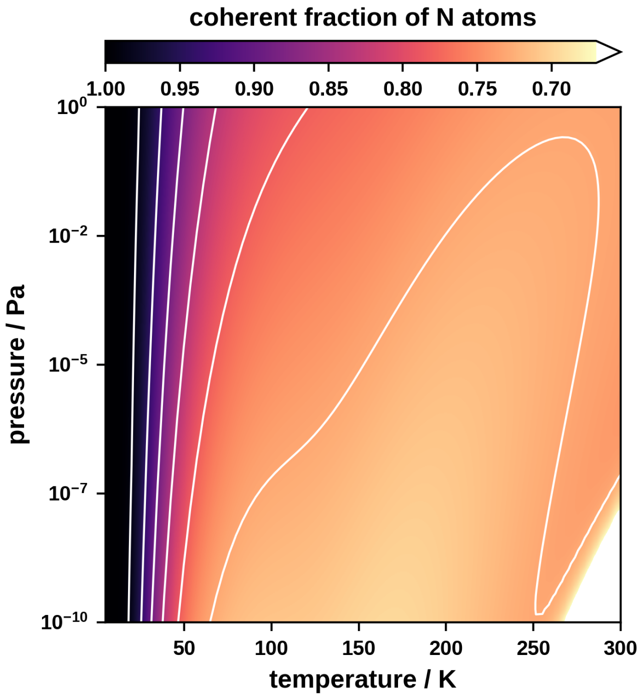

Figure S33: Thermodynamically populated phase diagram showing the expectation value of the coherent fraction for N atoms

Probability of Finding On-, Off- and Mixed-State Motifs

The thermal occupation yields a probability with which each individual structure occurs at a given temperature and pressure. Summing over all probabilities for a particular class of structure, say on-state structures, allows generating a plot that depicts the probability of finding any on-state structure. Figure S34 shows the probability (according to the thermal occupation) of finding on-state, off-state and mixed-state motifs plotted against the temperature at a constant pressure of 10^{-6} Pa.

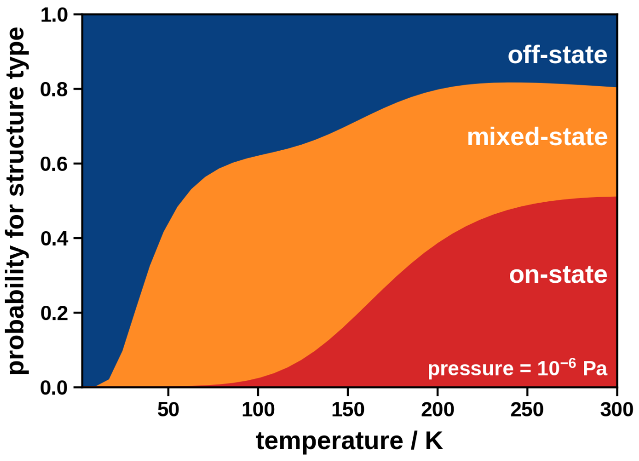

Figure S34: Diagram showing the probability of finding on-state, off-state and mixed-state motifs at different temperatures

As stated above the machine learning models we use have a prediction uncertainty for the adsorption energy. This uncertainty impacts how confident we can be about our results. Therefore, we will now gauge how much this uncertainty impacts our predictions. Our final prediction stems from the GPR-algorithm where the uncertainties are 0.039 $eV/molecule$ for on-state, 0.037 $eV/molecule$ for off-state and 0.033 $eV/molecule$ for mixed-state structures. Hence, we use $\sigma = 0.039$ $eV/molecule$ as the overall uncertainty of our predictions. To estimate the impact for this uncertainty we add normally distributed perturbations ΔE to the adsorption energies E_α.

$$E'_\alpha = E_\alpha + \Delta E, \;\; \Delta E \sim \mathcal{N}\left(0, \sigma^2\right) \qquad (10)$$

Then we use equation (7) to determine the thermal occupation (i.e. the probabilities for each structure to occur). We repeat this process 10^5 times and then determine the statistical mean value of the thermal occupation. This then allows determining the mean probability of finding on-, off- and mixed-state motifs. Figure S35 shows the mean probability of finding a particular type of structure.

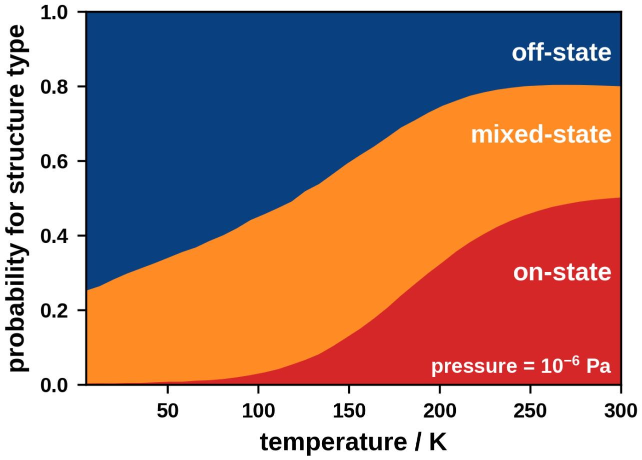

Figure S35: Diagram showing the probability, according to the statistical mean of the thermal occupation, of finding on-, off- and mixed-state motifs at different temperatures

We compare these mean probabilities to the most likely probabilities for finding a type of structure without accounting for the uncertainty (Figure S34). At 4 K there is a probability of approximately 30 % to find a mixed-state structure and a probability of approximately 70 % to find an off-state structure. Without the uncertainty the probability of an off-state structure is about 100 %. At 77 K there is a probability of approximately 40 % to find a mixed-state structure and a probability of approximately 55 % to find an off-state structure. Without the uncertainty the probability for finding a mixed-state structure is about 60 %. At room temperature the mean probability and the most likely probability are very similar. This is due to the fact that a large number of structure contributes to the thermal occupation at room temperature.

Comparison of Phase Diagrams

Besides using thermal occupation there are several other ways to generate phase diagrams from our data. These other methods are based, in part, on different assumptions and will therefore yield varying results. However, comparing these results allows gauging the robustness of our predictions.

The simplest option to construct a phase diagram is using the motifs with the lowest Gibbs free energy of adsorption at every temperature and pressure. This is shown in Figure S36. This method of analysis yields the largest range in $\Delta\Phi$ and coherent fraction. When switching between the mixed-state and the on-state interface we find a shift in $\Delta\Phi$ of almost 300 meV. Furthermore, the coherent fraction at 77 K and 300 K is approximately 0.4. While the trend is similar to what we observe for a thermal occupation (see Figure 3a and 3b in the main manuscript), the shifts are larger. Hence, the thermal occupation can be seen as a conservative estimate.

The second way to generate a phase diagram is using the statistical mean value of the thermal occupation with uncertainty, which we show in Figure S37. This phase diagram should be seen as a statistical tool of analysis rather than a representation of a real system. In a real system only one motif (if there are no degenerates) is thermally occupied a 0 K. The statistical mean of the thermal occupation, however, allows that a number of motifs contribute to the thermal occupation at 0 K. Most notably, here we no longer find a coherent fraction of 1.00 at 0 K. This is due to the statistical mean of the thermal occupation leading to mix of off- and mixed-state motifs even at low temperatures. On the other hand, the mixed-state and the on-state interface exhibit a very similar $\Delta\Phi$ and coherent fraction when compared to pristine thermal occupation (Figure 3a and 3b in the main manuscript).

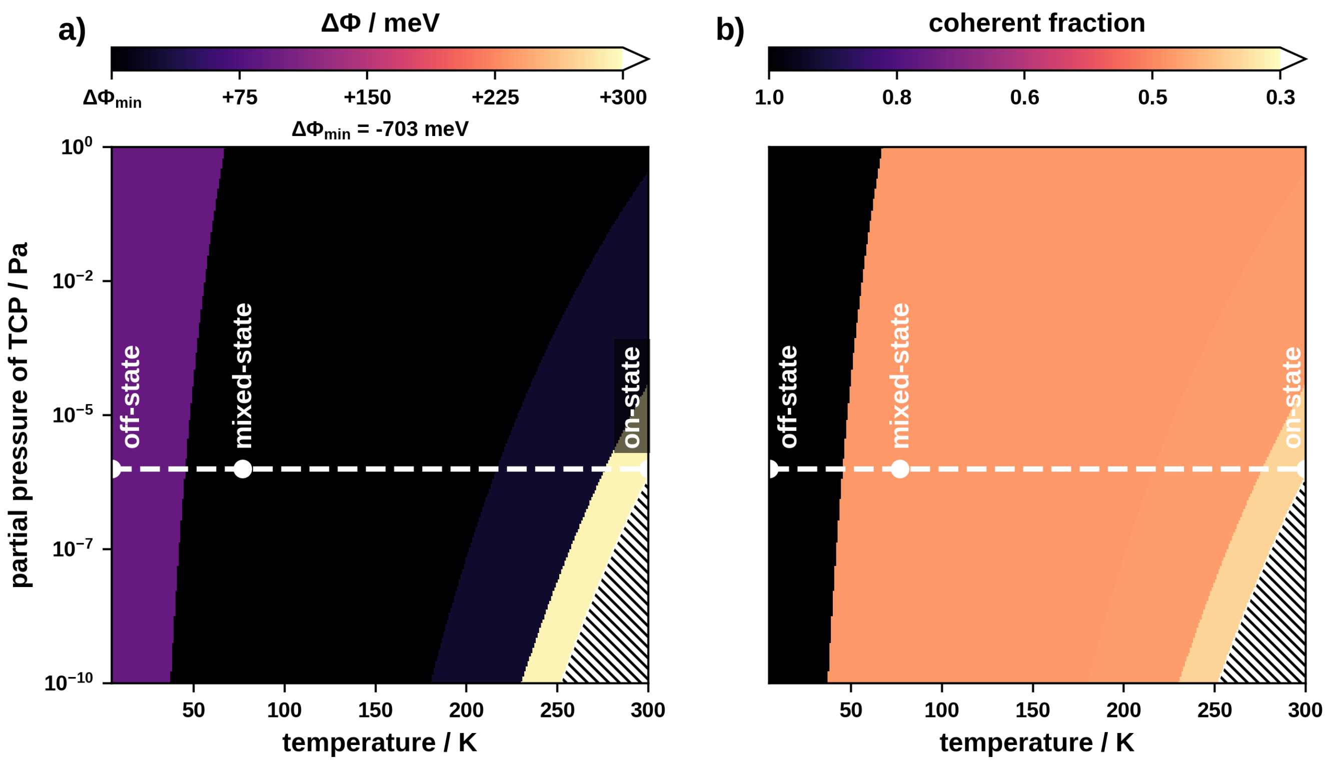

Figure S36: Phase diagram with (a) the $\Delta\Phi$ and (b) the coherent fraction of C atoms of the most energetically favourable structures, hatched areas indicate the thermodynamic range where adsorption is not energetically favourable

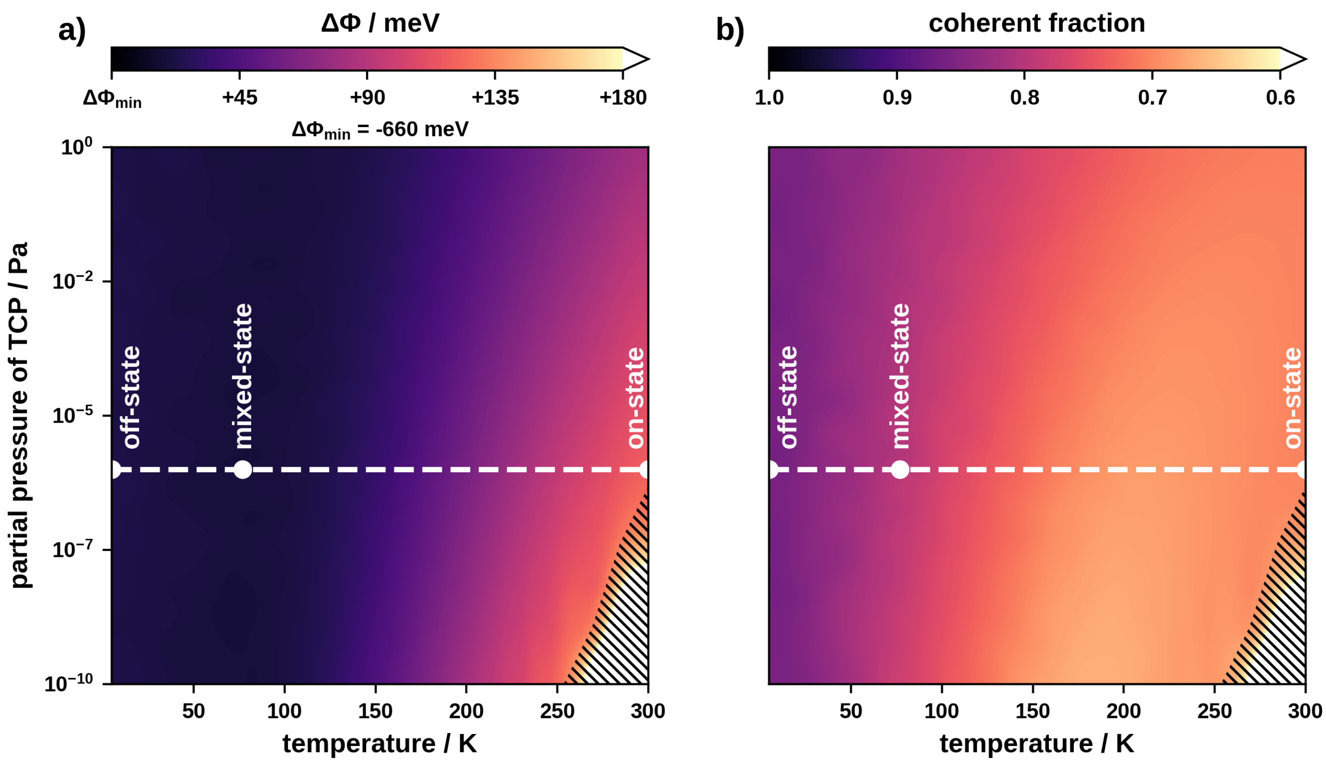

Figure S37: Phase diagram populated with a thermal occupation that accounts for the uncertainty. (a) expectation value of the $\Delta\Phi$, (b) expectation value of the coherent fraction of C atoms. Hatched areas indicate the thermodynamic range where adsorption is not energetically favourable

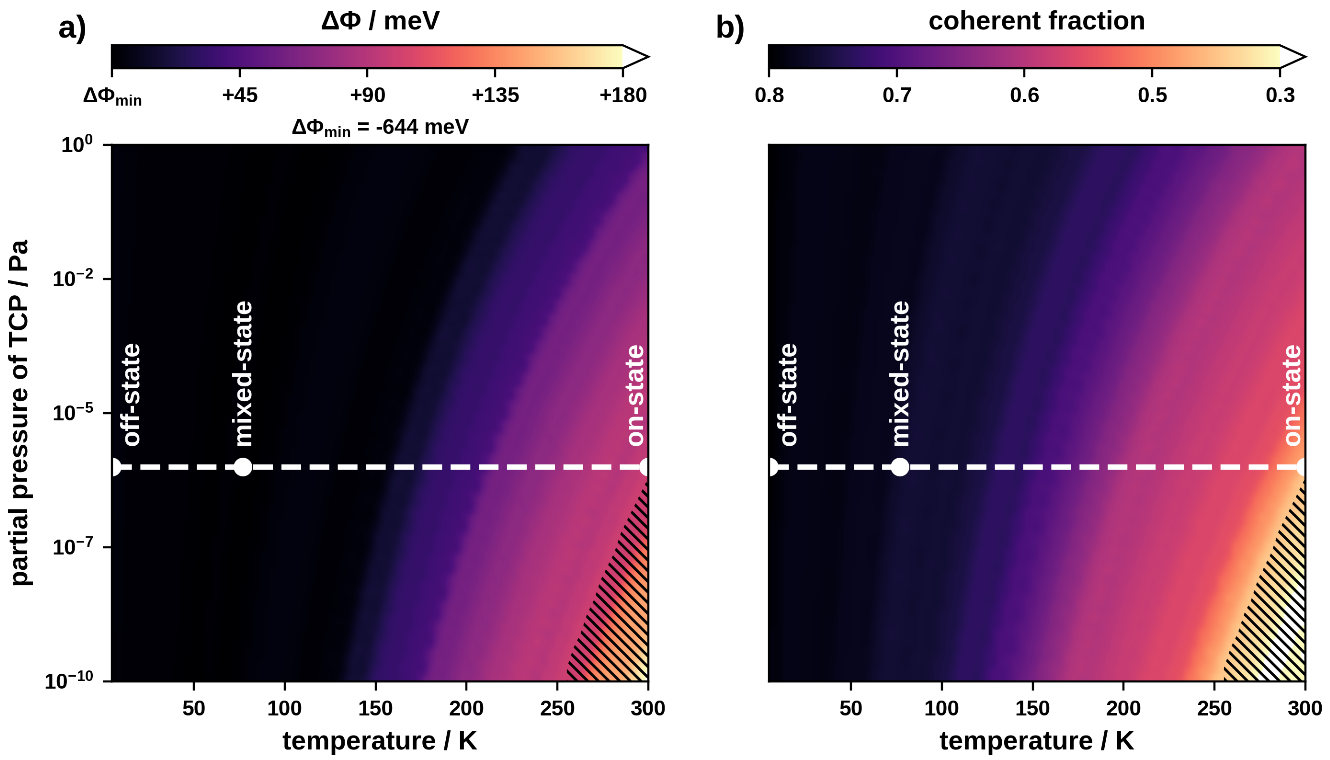

Figure S38: Phase diagram populated with (a) the mean $\Delta\Phi$ and (b) the mean coherent fraction of C atoms of structures that are within the uncertainty. Hatched areas indicate the thermodynamic range where adsorption is not energetically favourable

A third option to generate a phase diagram is using the mean value of all structures that are within the uncertainty of the adsorption energy prediction ($\approx 0.04eV$). Similar to the previous phase diagram, this one should be seen as a statistical tool of analysis. This phase diagram yields a phase diagram that is very similar to that using the statistical mean value of the thermal occupation. Here we also do not find a coherent fraction of 1.00 at 0 K. Other than that, the picture is qualitatively similar to the phase diagram based on the pristine thermal occupation (figure 3a and 3b in the main manuscript).

Charge Density Rearrangement and Dipole-Induced Potential Shifts

To determine the charge rearrangement resulting from the adsorption of TCP on the Pt(111) substrate we use the approach by Heimel et al..[6,7]

Figure S39 shows the rearrangement of charge density and the resulting potential shifts for the off-state geometries. Off-state geometries are very similar and exhibit only small rearrangements in charge density. This is expected, since off-state molecules interact mainly via vdW-interactions with the substrate. In a 4×4 surface unit cell the potential shift is approximately $-0.4\ eV$ (data shown for the most stable geometry).

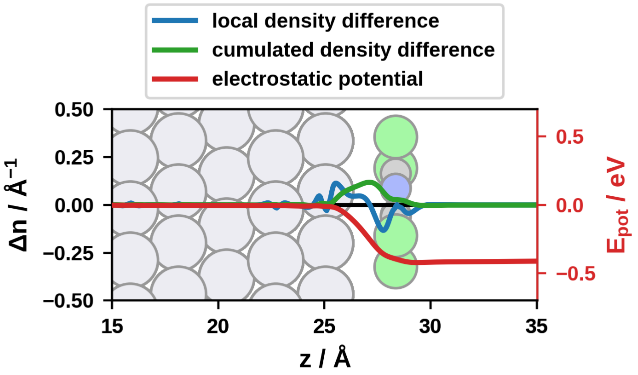

Figure S39: Plane integrated charge density differences and the resulting potential shifts for the off-state geometry

Conversely, on-state geometries can be separated into two significantly different groups: Three on-state geometries bind to the surface via N-atoms (N-bonded) while the fourth binds via C-atoms. Figures S40 and S41 depict rearrangements of charge density and resulting potential shifts for N-bonded and C-bonded geometries respectively. Due to hybridisation of the molecular states with the substrate, the local density in both cases shows notable fluctuations. The three N-bonded geometries exhibit a potential shift of approximately $-0.6\ eV$ in a 4×4 surface unit cell (data shown for the most stable N-bonded geometry). The C-bonded geometry has a much smaller potential shift of approximately $-0.2\ eV$.

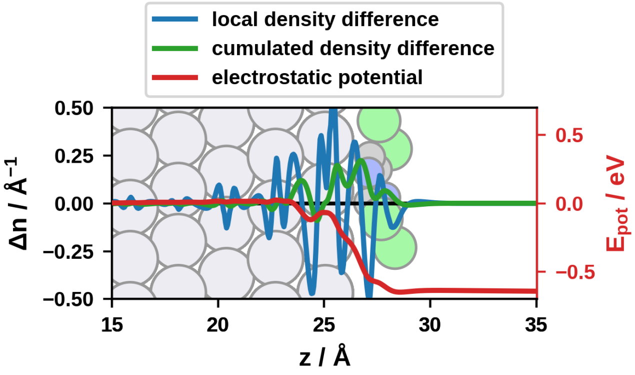

Figure S40: Plane integrated charge density differences and the resulting potential shifts for the N-bonded on-state geometry

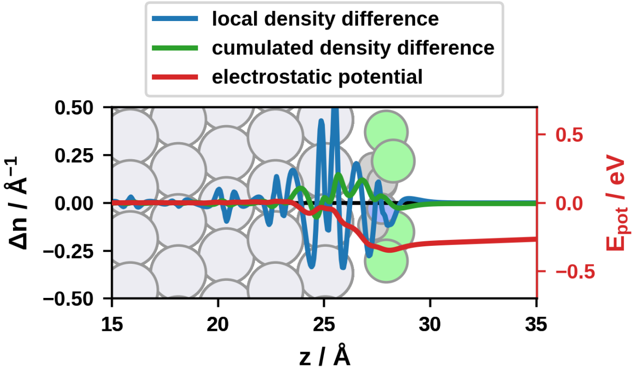

Figure S41: Plane integrated charge density differences and the resulting potential shifts for the C-bonded on-state geometry

These results coincide well with the work function changes we predict for close-packed adsorbate layers, which are in the range of $-0.7\ eV$ to $-0.4\ eV$.

References

1. Hörmann, L.; Jeindl, A.; Hofmann, O. T. Reproducibility of Potential Energy Surfaces of Organic/Metal Interfaces on the Example of PTCDA on Ag (111). *The Journal of Chemical Physics* **2020**, *153*, 104701.

2. Rogal, J.; Reuter, K. *Experiment, Modeling and Simulation of Gas-Surface Interactions for Reactive Flows in Hypersonic Flights*; RTO/NATO, 2007; pp 2–1 – 2–18.

3. Herrmann, P.; Heimel, G. Structure and Stoichiometry Prediction of Surfaces Reacting with Multicomponent Gases. *Advanced Materials* **2015**, *27*, 255–260.

4. Reuter, K.; Scheffler, M. Composition, Structure, and Stability of RuO_2 (110) as a Function of Oxygen Pressure. *Physical Review B* **2001**, *65*, 035406.

5. Jeindl, A.; Hörmann, L.; Hofmann, O. T. How Much Does Surface Polymorphism Influence the Work Function of Organic/Metal Interfaces? *Applied Surface Science* **2021**, 151687.

6. Heimel, G.; Romaner, L.; Zojer, E.; Brédas, J.-L. Toward control of the metal-organic interfacial electronic structure in molecular electronics: A first-principles study on self-assembled monolayers of π-conjugated molecules on noble metals. *Nano letters* **2007**, *7*, 932–940.

7. Heimel, G.; Romaner, L.; Brédas, J.-L.; Zojer, E. Interface energetics and level alignment at covalent metal-molecule junctions: π-conjugated thiols on gold. *Physical Review Letters* **2006**, *96*, 196806.
